# Supplementary material for: Engineering lentivirus envelope VSV-G for liver targeted delivery of IDOL-shRNA to ameliorate hypercholesterolemia and atherosclerosis
Source: Mol Ther Nucleic Acids. 2024 Jan 11;35(1):102115. doi: 10.1016/j.omtn.2024.102115 (PMC10835450; doi:10.1016/j.omtn.2024.102115)
Supplement: Document S1. Figures S1–S7 and Tables S1–S5 [file mmc1.pdf]

OMTN, Volume 35

Supplemental information

Engineering lentivirus envelope VSV-G for liver targeted delivery of IDOL-shRNA to ameliorate hypercholesterolemia and atherosclerosis

Wei Wang, Xuemei Chen, Jiali Chen, Menglong Xu, Ying Liu, Shijie Yang, Wenfeng Zhao, and Shuhua Tan

Supplemental Data

Supplemental Figures

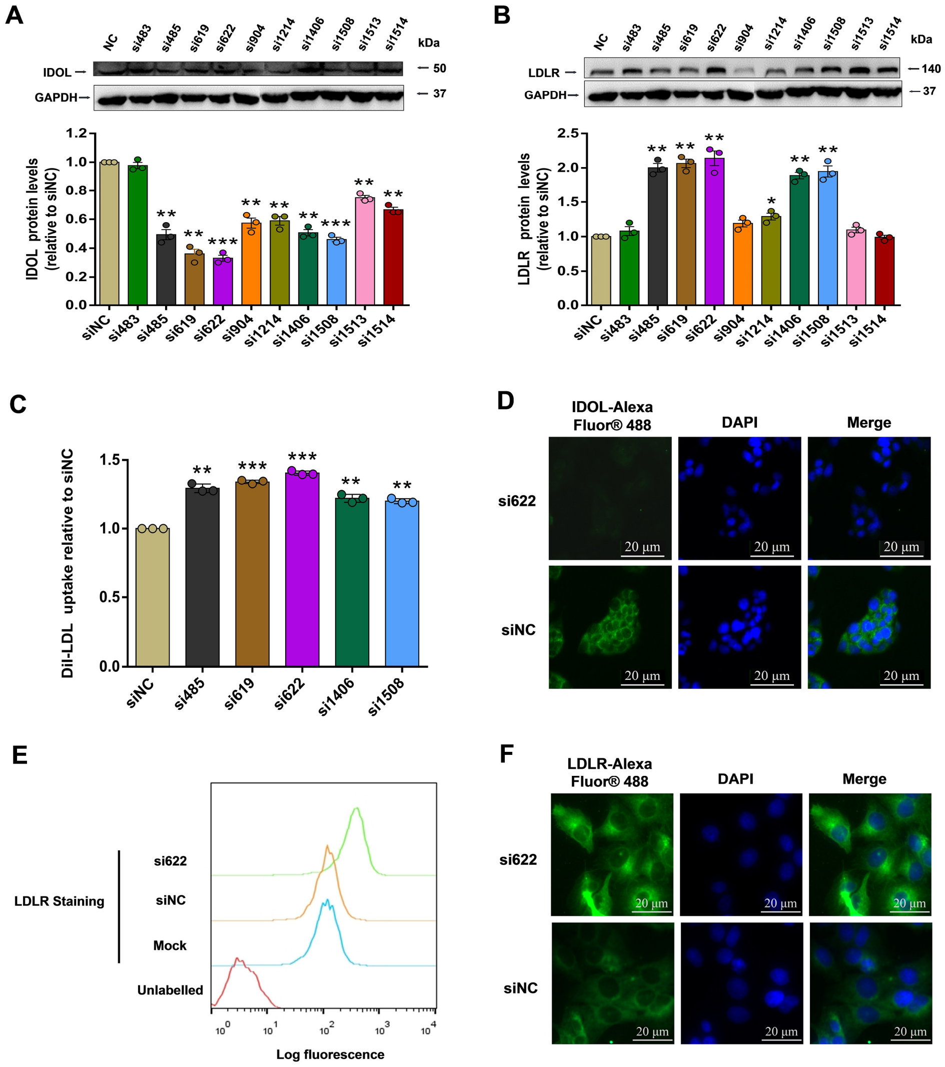

Fig. S1. Screening of IDOL siRNAs in human HepG2 hepatocytes. (**A** to **C**) Identification of efficacious IDOL siRNA in vitro. HepG2 cells were transfected with 30 nM siNC (negative control) and candidate siRNAs. 72h later, the levels of LDLR protein were analyzed by western blot normalized to that of the siNC group (**A** and **B**). After transfection with 30 nM siIDOL-485, siIDOL-619, siIDOL-622, siIDOL-1406 and siIDOL-1508 for 72 h, DiI-LDL uptake levels were measured using a Multimode Reader normalized to that of the siNC group (**C**). (**D** to **F**) Effects of siIDOL-622 on IDOL and LDLR expression in HepG2 cells. After transfection with 30 nM siNC and siIDOL-622 for 72h, IDOL protein levels were visualized by Immunofluorescence (**D**). The cell surface LDLR protein levels were determined by flow cytometry (**E**) and evaluated by Immunofluorescence (**F**). * p<0.05, ** p<0.01, *** p<0.001 vs siNC treatment group (unpaired Student's t-test). Results are given as the means±SEM of three independent experiments.

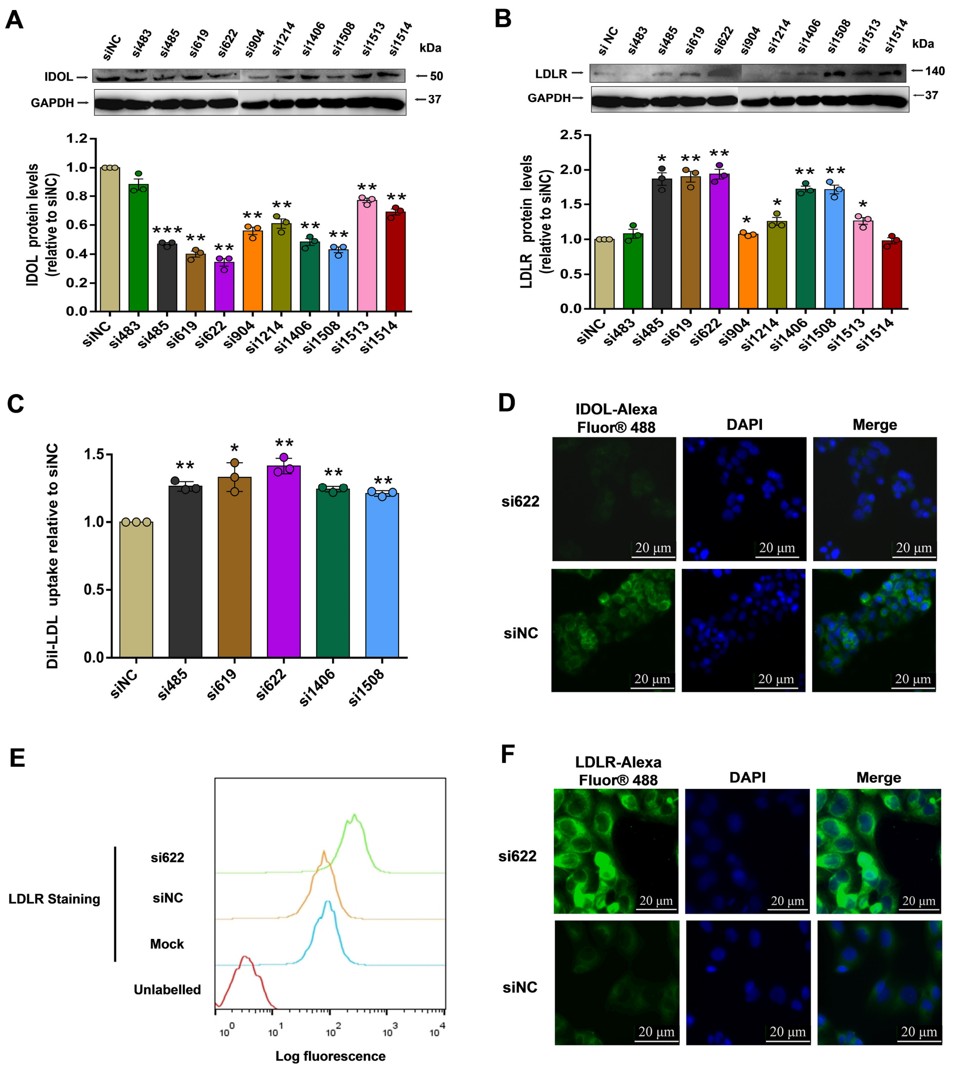

Fig. S2. Efficient siRNA-mediated knockdown of IDOL increases LDLR levels in mouse hepatic cells. (**A** and **B**) Hepa 1-6 cells were transfected with 30 nM siNC (negative control) and various IDOL siRNAs. 72h later, the levels of LDLR protein were analyzed by western blot normalized to that of the siNC group. (**C** to **F**) Effects of IDOL siRNAs on LDLR expression and DiI-LDL uptake in mouse hepatocytes. After transfection with 30 nM siIDOL-485, siIDOL-619, siIDOL-622, siIDOL-1406 and siIDOL-1508 for 72 h, DiI-LDL uptake levels were measured using a Multimode Reader normalized to that of the siNC group (**C**). Hepa 1-6 cells were transfected with 30 nM siNC and siIDOL-622. 72h later, protein levels of IDOL were evaluated by immunofluorescence (**D**). The cell surface LDLR protein levels were determined by flow cytometry (**E**) and visualized by Immunofluorescence (**F**). * $p<0.05$, ** $p<0.01$, *** $p<0.001$ vs siNC treatment group (unpaired Student's t-test). Results are given as the means±SEM of three independent experiments.

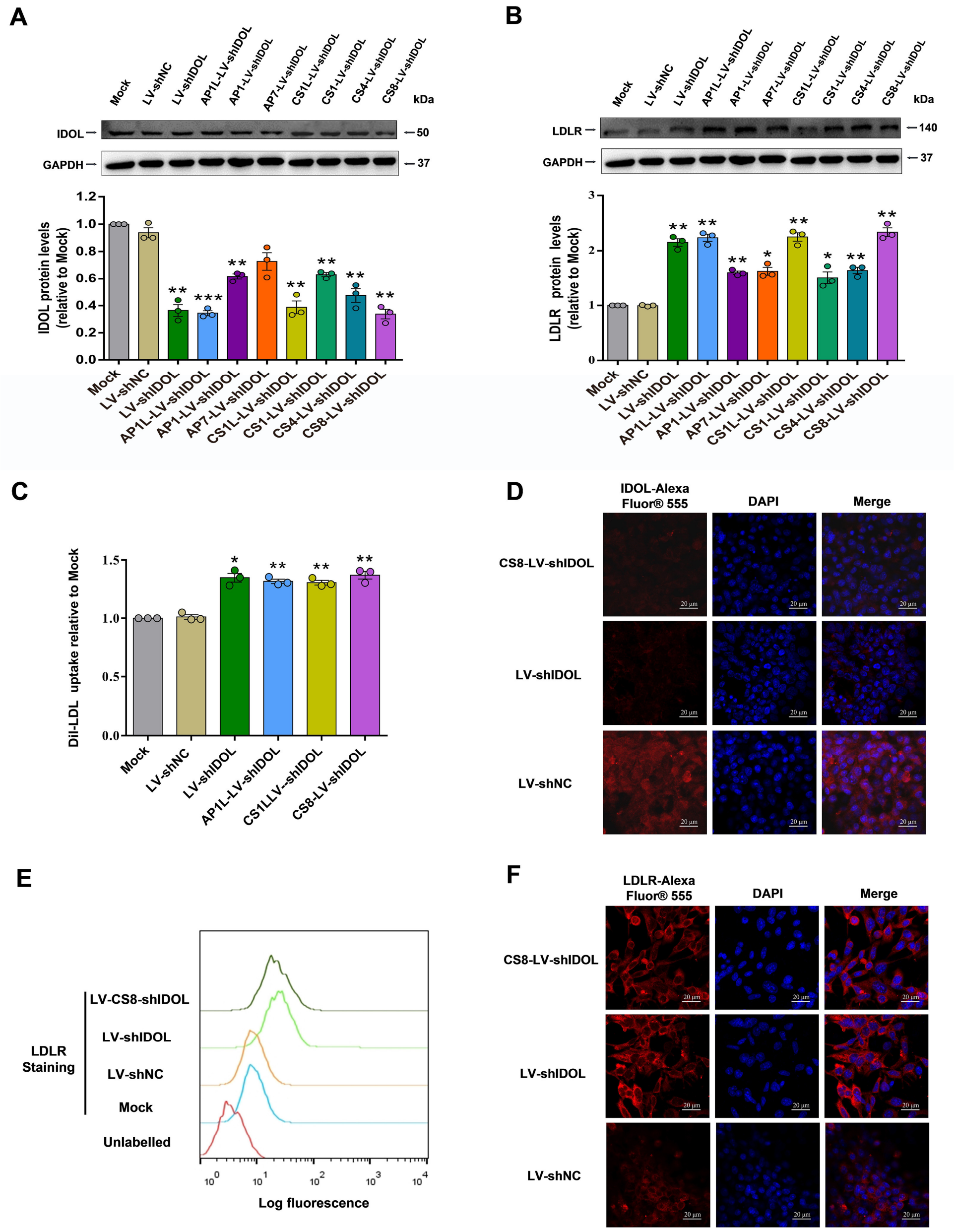

Fig. S3. Efficient shRNA-mediated silencing of IDOL elevates LDLR levels in mouse hepatocytes. (**A** and **B**) Hepa 1-6 cells were transducted with LV-shNC (negative control), LV-shIDOL (wild type control) and lentiviral IDOL shRNAs with different VSVG variants. 72h later, the levels of LDLR protein were analyzed by western blot normalized to that of the Mock group. (**C** to **F**) Effects of IDOL shRNAs on LDLR expression and DiI-LDL uptake in mouse hepatocytes. After transduction with LV-shIDOL, AP1L-LV-shIDOL, CS1L-LV-shIDOL and CS8-LV-shIDOL for 72 h, DiI-LDL uptake levels were measured using a Multimode Reader normalized to that of the Mock group (**C**). Hepa 1-6 cells were transducted with LV-shNC, LV-shIDOL and CS8-LV-shIDOL. 72h later, protein levels of IDOL were evaluated by immunofluorescence (**D**). The cell surface LDLR protein levels were determined by flow cytometry (**E**) and visualized by Immunofluorescence (**F**). * $p<0.05$, ** $p<0.01$, *** $p<0.001$ vs mock group (unpaired Student's t-test). Results are given as the means±SEM of three independent experiments.

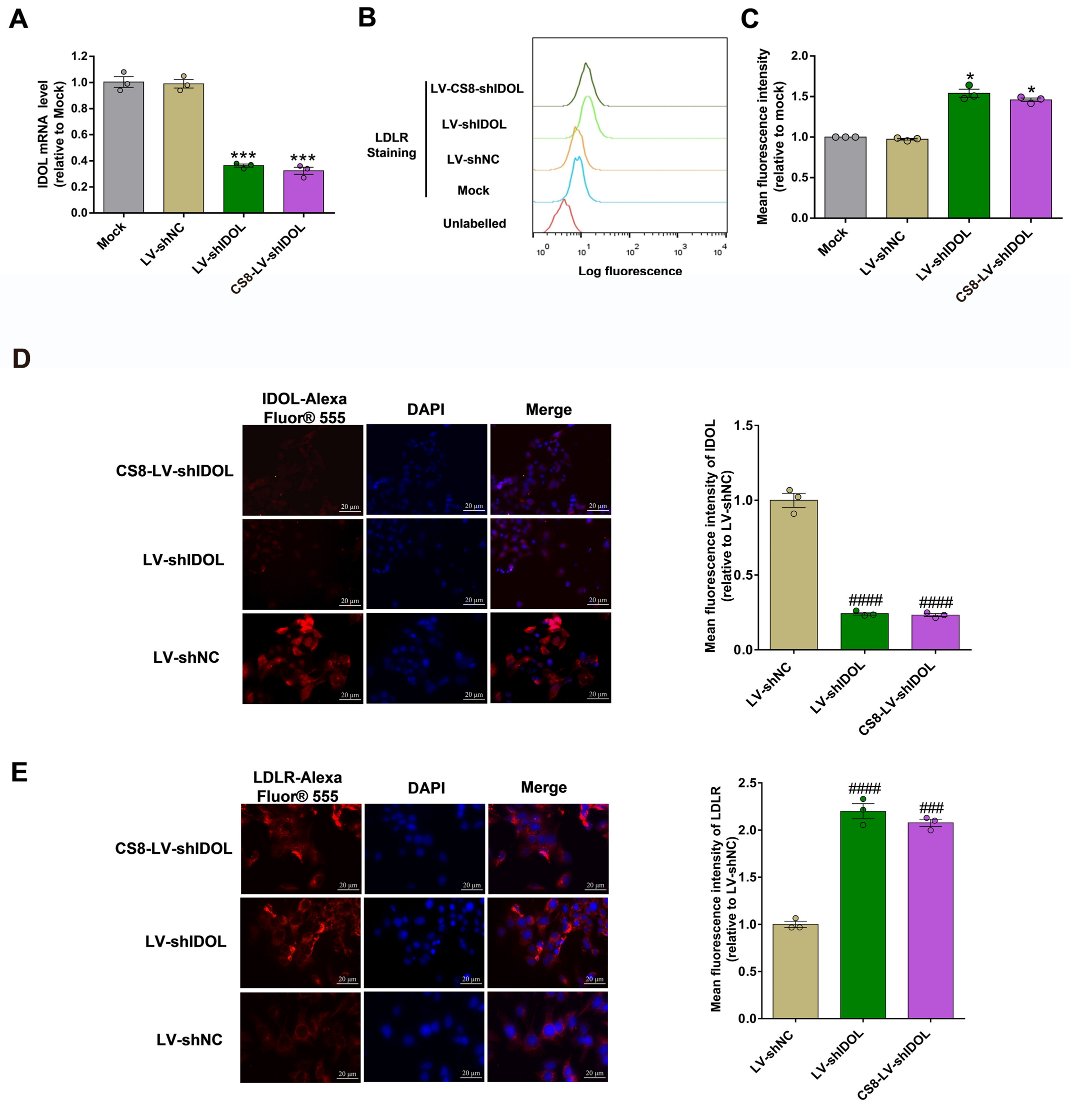

Fig. S4. Efficient shRNA-mediated silencing of IDOL elevates LDLR levels in LO2 cells. (**A**) LO2 cells were transducted with LV-shNC (negative control), LV-shIDOL (wild type control) and CS8-LV-shIDOL. After 48 h, the levels of IDOL mRNA in LO2 cells were quantifed by qRT-PCR. (**B-E**) Effects of IDOL shRNAs on LDLR expression in LO2 cells. LO2 cells were transducted with LV-shNC, LV-shIDOL and CS8-LV-shIDOL. 72h later, protein levels of IDOL were evaluated by immunofluorescence (**D**). The cell surface LDLR protein levels were determined by flow cytometry (**B,C**) and visualized by Immunofluorescence (**E**). * p<0.05, *** p<0.001 vs mock group; ### P< 0.001, #### P< 0.0001 vs. LV-shNC group (unpaired Student's t-test). Results are given as the means±SEM of three independent experiments.

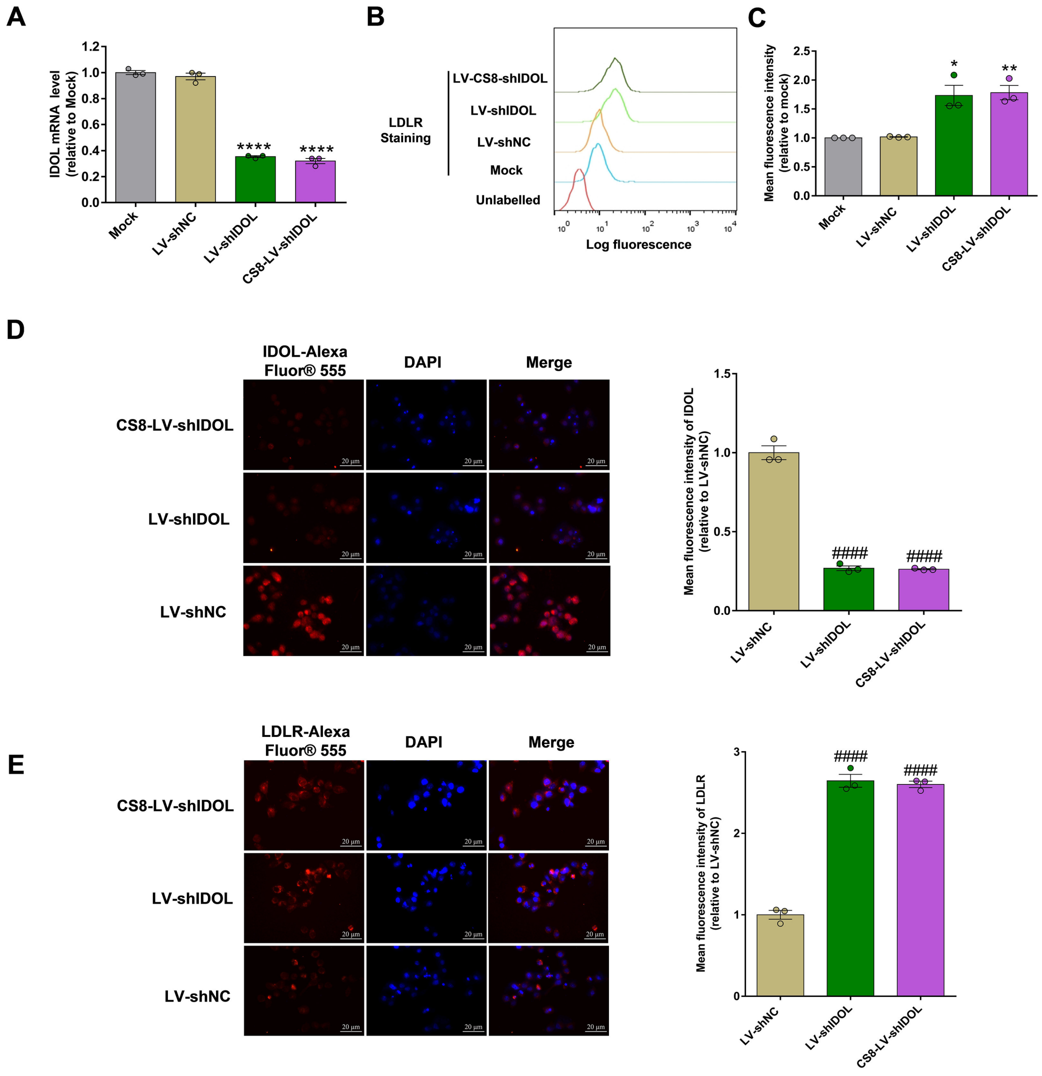

Fig. S5. Efficient shRNA-mediated silencing of IDOL elevates LDLR levels in AML12 cells. (**A**) AML12 cells were transducted with LV-shNC (negative control), LV-shIDOL (wild type control) and CS8-LV-shIDOL. After 48 h, the levels of IDOL mRNA in AML12 cells were quantifed by qRT-PCR. (**B-E**) Effects of IDOL shRNAs on LDLR expression in AML12 cells. AML12 cells were transducted with LV-shNC, LV-shIDOL and CS8-LV-shIDOL. 72h later, protein levels of IDOL were evaluated by immunofluorescence (**D**). The cell surface LDLR protein levels were determined by flow cytometry (**B,C**) and visualized by Immunofluorescence (**E**). * p<0.05, ** P<0.01, and **** p<0.0001 vs mock group; #### P< 0.0001 vs. LV-shNC group (unpaired Student's t-test). Results are given as the means±SEM of three independent experiments.

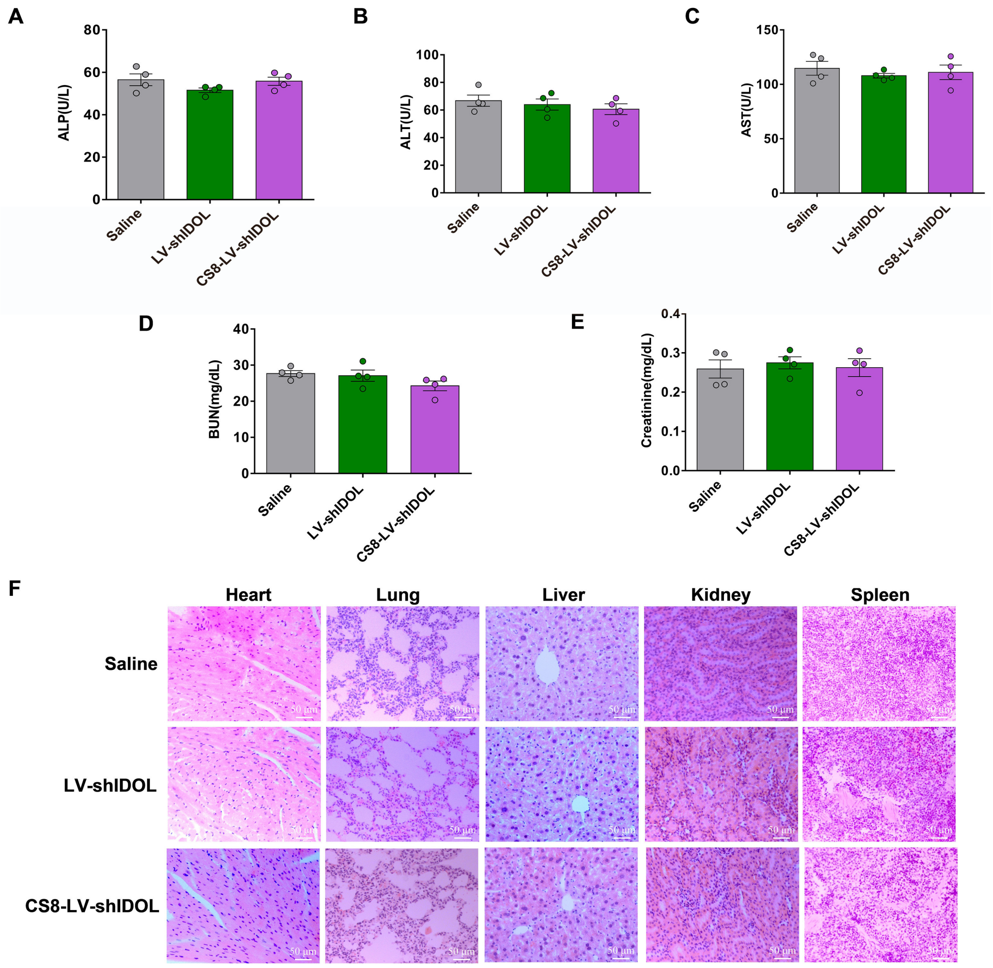

Fig. S6. In vivo biosafety analysis of CS8-LV-shIDOL. (**A-E**) Serum alanine aminotransferase (ALT), aspartate aminotransferase (AST), alkaline phosphatase (ALP), plasma urea (BUN) and creatinine were measured. Data represent mean values ± SEM. (F) Hematoxylin and eosin stainin images obtained from the major organs of LV-shIDOL, CS8-LV-shIDOL and saline treated mice. Scale bars, 50 µm.

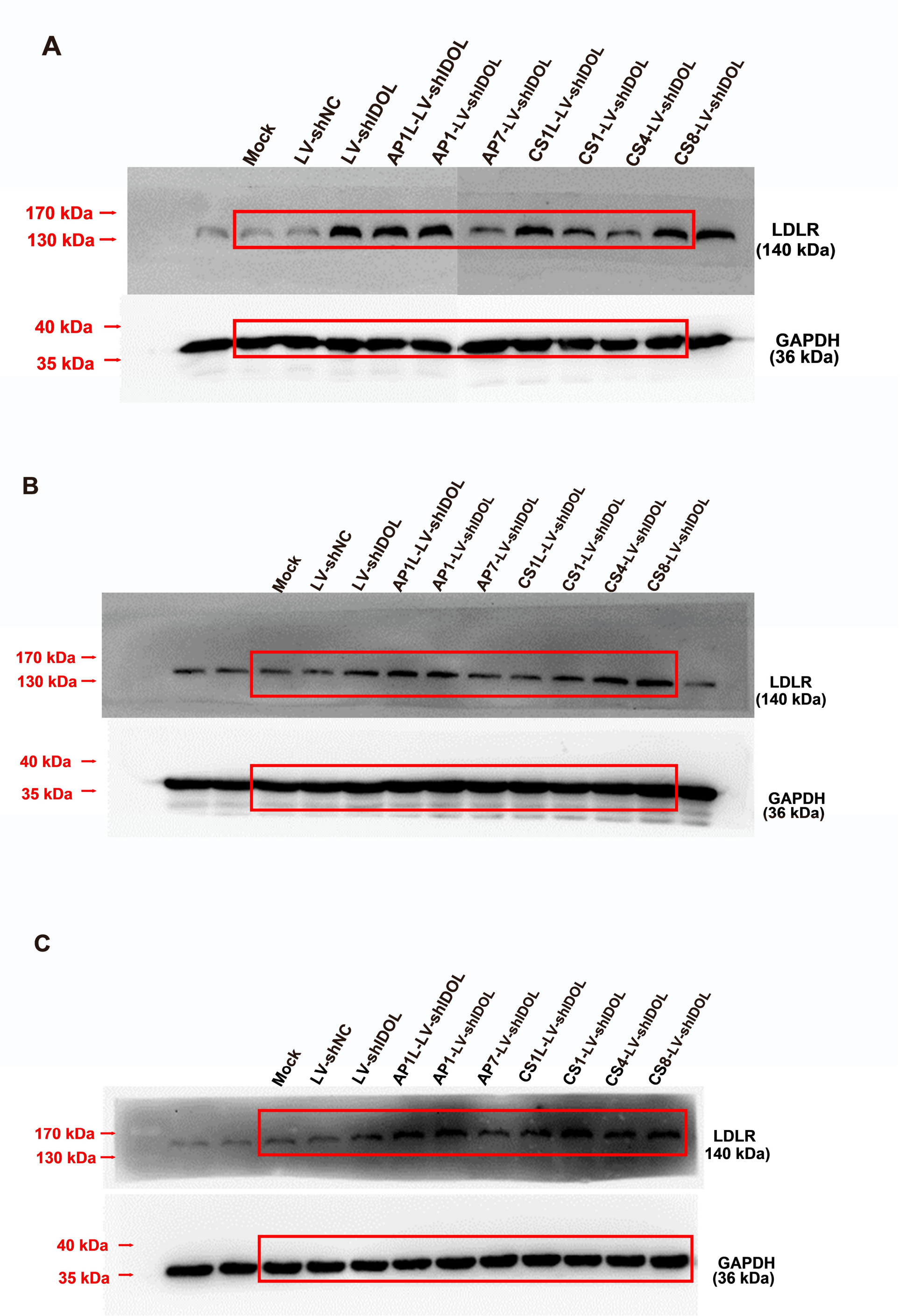

Fig. S7. Full scans of blots presented in cropped format for Figure 4C. Red boxes show the lanes included in the main text.

Supplemental Tables:

Table S1. Summary of various ligand insertion sites on VSV-G. The amino acid numbering used in this study was taken from that for the VSV-G crystal structure. Insertions were made into the wild type lentivirus envelope sequence, beginning immediately after the indicated VSV-G residue.

Name of constructs	Location	Ligand insertion site
AP1L	N-terminal	RLTRKRGLK-GGGGS-K1
AP1	N-terminal	RLTRKRGLK-K1
AP2	Domain I (Lateral domain)	Q10-RLTRKRGLK-K11
AP3	-	K50-RLTRKRGLK-A51
AP4	-	K174-RLTRKRGLK-G175
AP5	Domain III (PH domain)	G193-RLTRKRGLK-E194
AP6	Domain III (PH domain)	K200-RLTRKRGLK-E201
AP7	Domain III (PH domain)	E201-RLTRKRGLK-G202
AP8	Domain I (Lateral domain)	T351-RLTRKRGLK-T352
CS1L	N-terminal	HNMPNDPNRNVDENANANSAYC-GGGGS-K1
CS1	N-terminal	HNMPNDPNRNVDENANANSAYC-K1
CS2	Domain I (Lateral domain)	Q10-HNMPNDPNRNVDENANANSAYC-K11
CS3	-	K50-HNMPNDPNRNVDENANANSAYC-A51
CS4	-	K174-HNMPNDPNRNVDENANANSAYC-G175
CS5	Domain III (PH domain)	G193-HNMPNDPNRNVDENANANSAYC-E194
CS6	Domain III (PH domain)	K200-HNMPNDPNRNVDENANANSAYC-E201
CS7	Domain III (PH domain)	E201-HNMPNDPNRNVDENANANSAYC-G202
CS8	Domain I (Lateral domain)	T351-HNMPNDPNRNVDENANANSAYC-T352

Table S2. Nucleotide sequences of IDOL siRNAs used for screening in vitro. SiNC represents the negative control siRNA sequence. All the candidate siRNAs designed against different regions of human IDOL mRNA (Refseq ID: NM_013262.4).

Name	Sequence (5'-3')
siNC	UUCUCCGAACGUGUCACGU
siIDOL-483	GGAGCCUCAUCUCAUCUUA
siIDOL-485	AGCCUCAUCUCAUCUUACA
siIDOL-619	UUUGGAGACUACAACCAGA
siIDOL-622	GGAGACUACAACCAGAACA
siIDOL-904	GCUUAUCCUGUGGUGCAGA
siIDOL-1214	CCAGGAGGGCUCUGUACAA
siIDOL-1406	GCUGCGAGGAGGAGAUCAA
siIDOL-1508	GUGUGGAGCAUGUCCAGCA
siIDOL-1513	GAGCAUGUCCAGCACGUCU
siIDOL-1514	AGCAUGUCCAGCACGUCUA

Table S3. Nucleotide sequences of IDOL shRNAs used for the lentiviral vector construction. ShNC represents the negative control shRNA sequence and shIDOL is designed based on the sequence of siIDOL-622. The bold letters represent the sequences of negative control siRNA and siIDOL-622.

Name		Sequence (5'→ 3')
shNC	sense	GATCC**GCGACGATCTGCCTAAGAT**TTCAAGAGA**ATCTTAGGCAGATCGTCGC**TTTTTTG
	antisense	AATTCAAAAAA**GCGACGATCTGCCTAAGAT**TCTCTTGAA**ATCTTAGGCAGATCGTCGC**G
shIDOL	sense	GATCC**GGAGACTACAACCAGAACA**TTCAAGAGA**TGTTCTGGTTGTAGTCTCC**TTTTTTG
	antisense	AATTCAAAAAA**GGAGACTACAACCAGAACA**TCTCTTGAA**TGTTCTGGTTGTAGTCTCC**G

Table S4. Primer list for construction of lentivrus envelope mutant plasmids.

Primer	Sequence (5'→3')
HF	CCCAAGCTTACATGTGGTACCGAGCTCGGA
PR	CCAATGCATTGGTTCTGCAGCAAAGAGATCCTTATC
PF	AAAACTGCAGCCAGATTCCCTGAATGCC
NR	TTTTCCTTTTGCGGCCGCACTAGTGCTAGACTGCCA
AP1F	TTGACAAGAAAAAGGGGATTGAAGAAGTTCACCATAGTTTTTCC
AP1R	CAATCCCCTTTTTCTTGTCAATCTGCAATTCACCCCAATGAATA
AP1LF	AAGGGCGGCGGCAGCAAGTTCACCATAGTTTTTCCACAC
AP1LR	CTTGCTGCCGCCGCCGCCCTTCAATCCCCTTTTTCTTGTCAA
AP2F	TTGACAAGAAAAAGGGGATTGAAGAAAGGAAACTGGAAAAATGT
AP2R	CAATCCCCTTTTTCTTGTCAATCTTTGGTTGTGTGGAAAAACTA
AP3F	TTGACAAGAAAAAGGGGATTGAAGGCTATTCAAGCAGACGGTTG
AP3R	CAATCCCCTTTTTCTTGTCAATCTCTTGTGACTCTTGGGCATTT
AP4F	TTGACAAGAAAAAGGGGATTGAAGGGGCTATGTGATTCTAACCT
AP4R	CAATCCCCTTTTTCTTGTCAATCTTTTGACCTTATAGTCAGAAT
AP5F	TTGACAAGAAAAAGGGGATTGAAGGAGCTATCATCCCTGGGAAA
AP5R	CAATCCCCTTTTTCTTGTCAATCTTCCGTCCTCTGAGA
AP6F	TTGACAAGAAAAAGGGGATTGAAGGAGGGCACAGGGTTCAGAA
AP6R	CAATCCCCTTTTTCTTGTCAATCTCTTTCCCAGGGATGATAGC
AP7F	TTGACAAGAAAAAGGGGATTGAAGGGCACAGGGTTCAGAAGTAA
AP7R	CAATCCCCTTTTTCTTGTCAATCTCTCCTTTCCCAGGGATGATA
AP8F	TTGACAAGAAAAAGGGGATTGAAGACAGAAAGGGAACTGTGGGA
AP8R	CAATCCCCTTTTTCTTGTCAATCTGGTAGTTCCACTGATCATTC
CS1F	AACCGAAATGTAGATGAAAATGCTAATGCCAACAGTGCTAAGTTCAC CATAGTTTTTCC
CS1R	ATTTTCATCTACATTTCGGTTTGGGTCATTTGGCATATTGTGGCAATTC ACCCCAATGA
CS1LF	GCTGGCGGCGGCGGCAGCAAGTTCACCATAGTTTTTCCACAC
CS1LR	CTTGCTGCCGCCGCCGCCAGCACTGTTGGCATTAGCATTTTC
CS2F	AACCGAAATGTAGATGAAAATGCTAATGCCAACAGTGCTAAAGGAAA

	CTGGAAAAATGT
CS2R	ATTTTCATCTACATTTCGGTTTGGGTCATTTGGCATATTGTGTTGGTTG TGTGGAAAAA
CS3F	AACCGAAATGTAGATGAAAATGCTAATGCCAACAGTGCTGCTATTCA AGCAGACGGTTG
CS3R	ATTTTCATCTACATTTCGGTTTGGGTCATTTGGCATATTGTGCTTGTGA CTCTTGGGC
CS4F	CCAAACCGAAATGTAGATGAAAATGCTAATGCCAACAGTGCTGGGCT ATGTGATTCTAA
CS4R	ATTTTCATCTACATTTCGGTTTGGGTCATTTGGCATATTGTGTTTGACC TTATAGTCAG
CS5F	CCAAACCGAAATGTAGATGAAAATGCTAATGCCAACAGTGCTGAGCT ATCATCCCTGG
CS5R	ATTTTCATCTACATTTCGGTTTGGGTCATTTGGCATATTGTGTCCGTCC TCTGAGAAGA
CS6F	AACCGAAATGTAGATGAAAATGCTAATGCCAACAGTGCTGAGGGCAC AGGGTTCAGAAG
CS6R	ATTTTCATCTACATTTCGGTTTGGGTCATTTGGCATATTGTGCTTTCCC AGGGATGATA
CS7F	CCAAACCGAAATGTAGATGAAAATGCTAATGCCAACAGTGCTGGCAC AGGGTTCAGAAG
CS7R	ATTTTCATCTACATTTCGGTTTGGGTCATTTGGCATATTGTGCTCCTTTC CCAGGGAT
CS8F	CCAAACCGAAATGTAGATGAAAATGCTAATGCCAACAGTGCTACAGA AAGGGAACTGTG
CS8R	ATTTTCATCTACATTTCGGTTTGGGTCATTTGGCATATTGTGGGTAGTT CCACTGATCA

Table S5. Primers for qRT-PCR.

Gene	Primer sequence (5'-3')	
	Forward	Reverse
Human IDOL	CACCCAGTCAGGAAAG	GTTATCGCTCGGTAGAG
Human GAPDH	TGACAACAGCCTCAAGAT	ACCACCCTGTTGCTGTAGCC
Mouse IDOL	CCCAGTCAGGAAAGAAT	GGTGATGGCTCGGTAG
Mouse β-actin	GTGACGTTGACATCCGTAAAGA	GCCGGACTCATCGTACTCC
