# Supplementary material for: Engineering lentivirus envelope VSV-G for liver targeted delivery of IDOL-shRNA to ameliorate hypercholesterolemia and atherosclerosis
Source: Mol Ther Nucleic Acids. 2024 Jan 11;35(1):102115. doi: 10.1016/j.omtn.2024.102115 (PMC10835450; doi:10.1016/j.omtn.2024.102115)
Supplement: Document S2. Article plus supplemental information [file mmc2.pdf]

Molecular Therapy
Nucleic Acids
Original Article

# Engineering lentivirus envelope VSV-G for liver targeted delivery of IDOL-shRNA to ameliorate hypercholesterolemia and atherosclerosis

Wei Wang,[1,2] Xuemei Chen,[1,2] Jiali Chen,[1] Menglong Xu,[1] Ying Liu,[1] Shijie Yang,[1] Wenfeng Zhao,[1] and Shuhua Tan[1]

[1]Department of Cell and Molecular Biology, School of Life Science and Technology, State Key Laboratory of Natural Medicines, Jiangsu Key Laboratory of Druggability of Biopharmaceuticals, China Pharmaceutical University, Nanjing 210009, China

**Lentiviral vectors (LVs) have been widely used as a tool for gene therapies. However, tissue-selective transduction after systemic delivery remains a challenge. Inducible degrader of low-density lipoprotein receptor is an attractive target for treating hypercholesterolemia. Here, a liver-targeted LV, CS8-LV-shIDOL, is developed by incorporating a hepatocyte-targeted peptide derived from circumsporozoite protein (CSP) into the lentivirus envelope for liver-targeted delivery of IDOL-shRNA (short hairpin RNA) to alleviate hypercholesterolemia. Tail-vein injection of CS8-LV-shIDOL results in extremely high accumulation in liver and nearly undetectable levels in other organs in mice. In addition, it shows superior therapeutic efficacy in lowering serum low-density lipoprotein cholesterol (LDL-C) and reducing atherosclerotic lesions over unmodified LV-shIDOL in hyperlipidemic mice. Mechanically, the envelope-engineered CS8-LV-shIDOL can enter liver cells via low-density lipoprotein receptor-related protein (LRP). Thus, this study provides a novel approach for liver-targeted delivery of IDOL-shRNA to treat hypercholesterolemia by using an envelope-engineered LV, and this delivery system has great potential for liver-targeted transgene therapy.**

## INTRODUCTION

Lentiviral vectors (LVs) have been widely used as a tool for gene and cell therapies, and the number of gene therapy clinical trials using LVs is increasing.[1,2] To date, two lentivirus-based *ex vivo* gene therapies, Kymriah (Novartis) and Zynteglo (bluebird bio), have been approved.[3] Besides, direct *in vivo* applications of lentivirus for treating patients with Parkinson's disease and age-related macular degeneration (AMD) have been reported.[4,5] Compared with other retrovirus vectors, LVs possess several major advantages, which include efficient transduction of nondividing cells, low cytotoxicity and immunogenicity, relatively large transgene capacity of approximately 8–9 kb, and long-term transgene expression.[6,7] Notably, LVs are commonly pseudotyped with the vesicular stomatitis virus envelope glycoprotein (VSV-G), which confers not only high particle stability and high viral titers but also broad cell tropism to the LVs, while the wide cell tropism can lead to the binding of VSV-G pseudotyped LVs to the surface of any cell encountered before reaching the target cells.[8] Hence, improving the selective transduction of LVs to the target cells after systemic delivery remains a challenge.

As the crystal structure of VSV-G has been determined,[9,10] it is feasible to rationally design and engineer VSV-G to adapt the lentiviral tropism to the particular target cells. Previously, it has been identified that the circumsporozoite protein (CSP) of *Plasmodium falciparum* plays an important role in sporozoite entry into hepatocytes,[11,12] which is attributed to the specific binding of *P. falciparum* CSP peptides to hepatocytes.[13] Similarly, two peptide segments derived from ApoB-100 have been shown to mediate the binding of low-density lipoprotein (LDL) to the LDL receptor (LDLR).[14] Thus, we hypothesize that these peptides can be potentially exploited as the targeting ligands for engineering VSV-G to restrict LV entry to liver cells.

The inducible degrader of LDLR, also known as MYLIP, is an unique E3 ubiquitin ligase[15] that directly interacts with the cytoplasmic tail of LDLR and facilitates its ubiquitination and subsequent degradation in lysosome,[16,17] thus decreasing hepatic LDLR levels and reducing hepatic LDL uptake.[18,19] Hence, IDOL may serve as a promising therapeutic target for treating dyslipidemia and atherosclerotic cardiovascular disease (ASCVD).[20–22] Recently, a cyclic peptide has been shown to dose-dependently increase LDLR levels via inhibiting the homodimerization of IDOL E3 ubiquitin ligase in hepatic cells.[23]

RNAi therapeutics have received tremendous attention as an inhibitor to reduce the expression of disease associated proteins in patients.[24–27] Thus, developing RNAi therapeutics to suppress hepatic IDOL expression appears to be an alternative approach to treat hypercholesterolemia. Yet how to efficiently and selectively deliver

Received 12 August 2023; accepted 5 January 2024;
https://doi.org/10.1016/j.omtn.2024.102115.

[2]These authors contributed equally

**Correspondence:** Shuhua Tan, Department of Cell and Molecular Biology, School of Life Science and Technology, State Key Laboratory of Natural Medicines, Jiangsu Key Laboratory of Druggability of Biopharmaceuticals, China Pharmaceutical University, Nanjing 210009, China.
**E-mail:** 1019972185@cpu.edu.cn


This is an open access article under the CC BY license (http://creativecommons.org/licenses/by/4.0/).

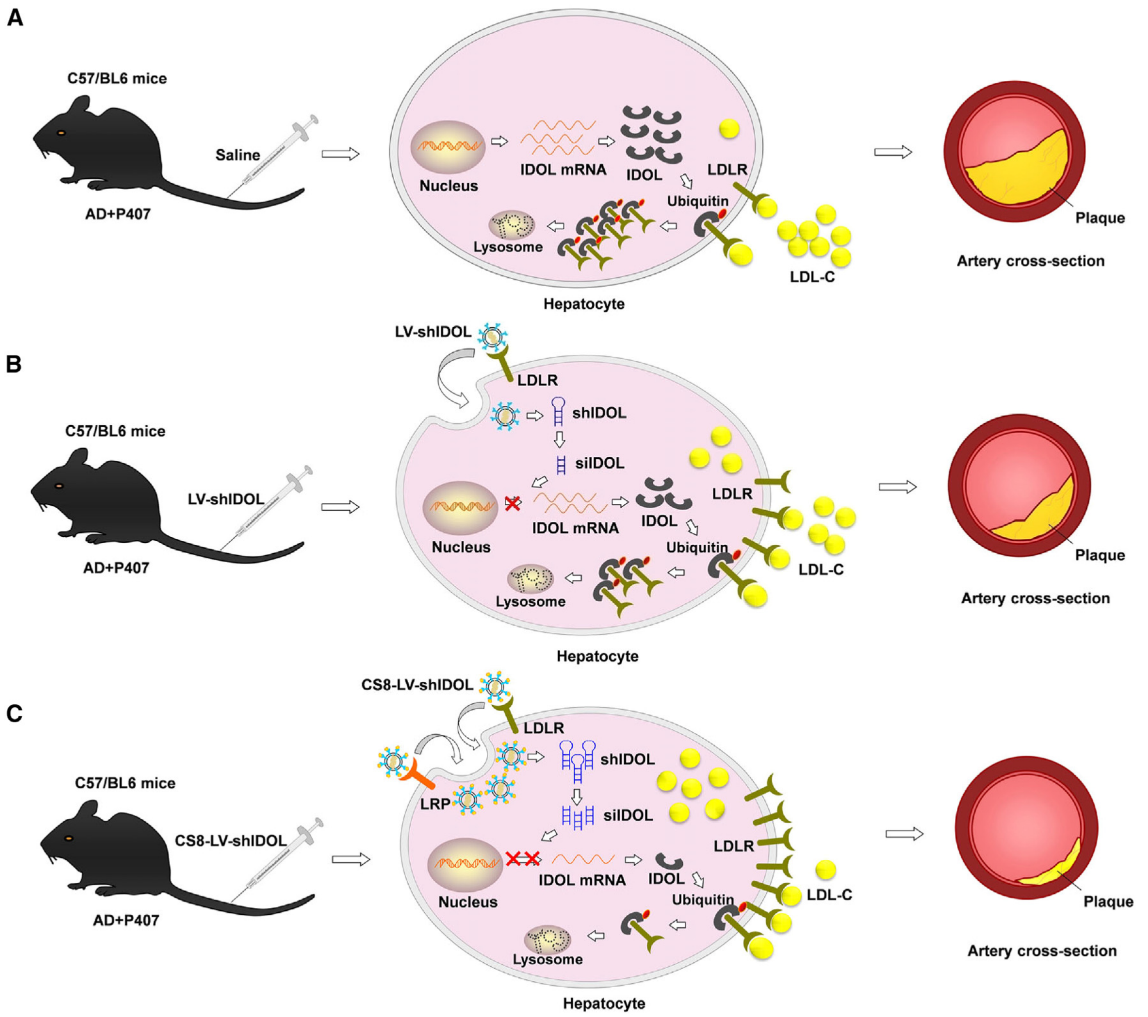


**Figure 1. Schematic diagram illustrating the mechanism of liver-targeted lentiviral vector CS8-LV-shIDOL treating atherosclerosis in C57/BL6 mice**
(A) P407-induced hyperlipidemic mice fed an atherogenic diet (AD) treated with saline. IDOL binds to and promotes ubiquitination of the intracellular tail of the LDLR, resulting in lysosomal degradation of the receptor. The aortas of P407-induced atherogenic mice show pathological lesions that are thickened. (B) P407-induced hyperlipidemic mice fed an AD treated with wild-type lentiviral vector LV-shIDOL. Lentiviral vector mediated shRNA knockdown of IDOL up-regulates LDLR and lowers circulating LDL levels. The lesions of the aorta in LV-shIDOL-treated atherogenic mice are milder than those in untreated atherogenic mice. (C) P407-induced hyperlipidemic mice fed an AD treated with liver-targeted CS8-LV-shIDOL. Compared with LV-shIDOL, liver-targeted CS8-LV-shIDOL has higher potency in inhibiting hepatic IDOL *in vivo*, resulting in higher LDLR levels and lower serum LDL levels. The lesions of the aorta in CS8-LV-shIDOL-treated atherogenic mice are milder than those in LV-shIDOL-treated atherogenic mice.

therapeutic small interfering RNAs (siRNAs) against IDOL to hepatocytes after systemic delivery needs to be addressed. In the present study, we sought to develop a liver-targeted LV by engineering the envelope of lentivirus to facilitate targeted delivery of IDOL-shRNA (short hairpin RNA) to liver to more effectively ameliorate hypercholesterolemia and atherosclerosis (Figure 1), while also providing a potential lentivirus delivery system for liver-targeted transgene therapy.

## RESULTS

### Rational design and engineering of a liver-targeted LV system

Two hepatocyte-targeted peptides were selected as targeting ligands for engineering VSV-G protein. One was a 9 amino acid peptide (RLTRKRGLK) derived from ApoB-100 segments (AP),[14] and the other was a 20 amino acid peptide (HNMPNDPNRNV DENANANSAYC) derived from *P. falciparum* CSP.[13] Meanwhile, the prefusion form of VSV-G crystal structure was used to explore the feasible insertion sites.[10] The amino acid sequences of glycoproteins G from various vesicular stomatitis virus serotypes were aligned and analyzed to find potential sites that might tolerate insertion of a foreign peptide. As shown in Figure 2, multiple potential insertion sites in VSV-G were identified on the basis of three criteria: flexible loop regions, unconserved regions, and regions rich in hydrophilic amino acids.[28,29] Here, the amino acid was numbered referring to the literature elucidating VSV-G crystal

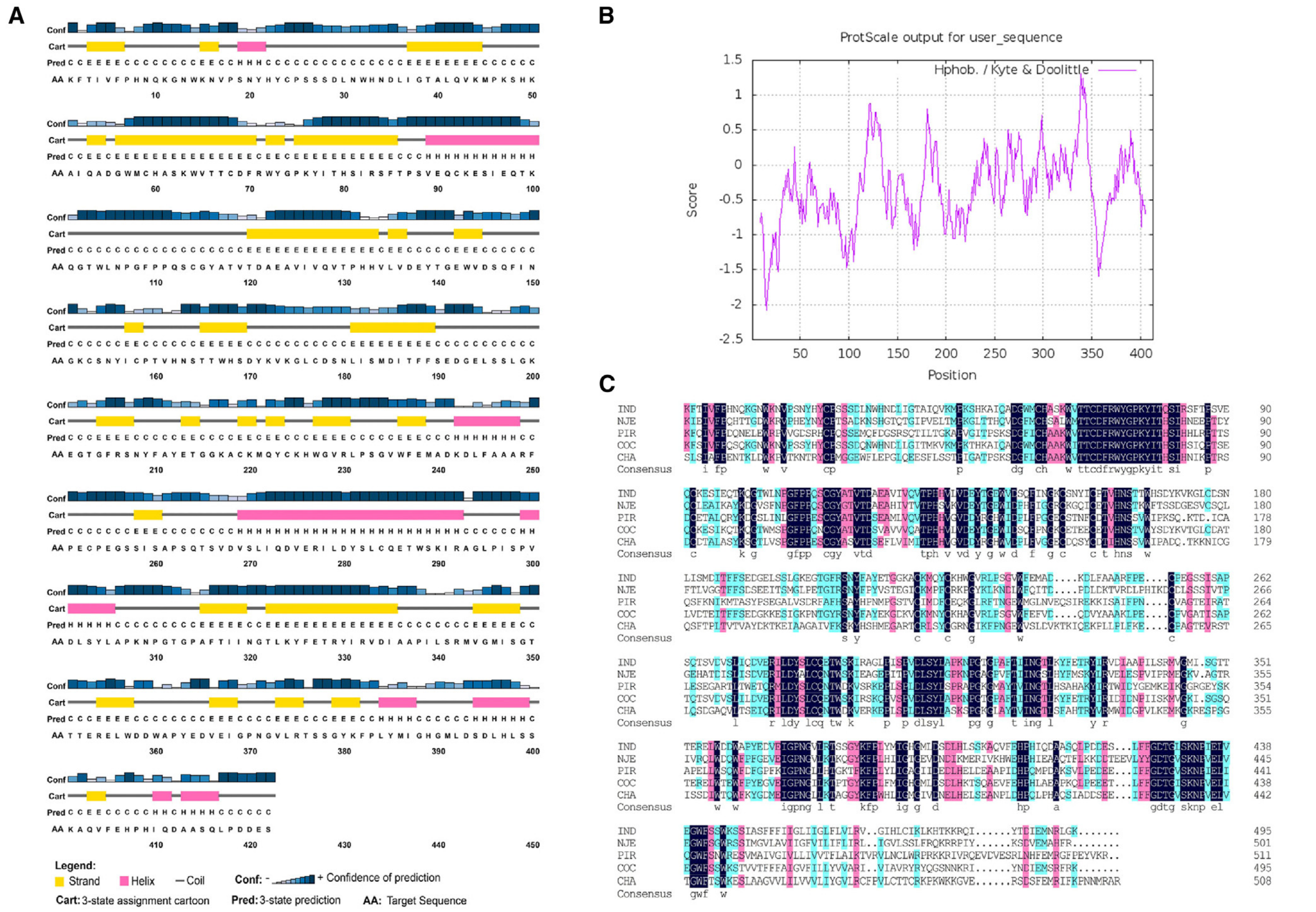


**Figure 2. Analysis on viable insertion sites on VSV-G for displaying hepatocyte-targeting peptides**

(A) Elements of secondary structure are indicated in the upper of the amino acid sequence of VSV-G. The helices are shown in the pink rectangle, the strands in the yellow rectangle, and the coiled coils as a gray line. The secondary structure of VSV-G was analyzed using PSIPRED server (http://bioinf.cs.ucl.ac.uk/psipred/). (B) Hydrophobicity plot corresponding to sequences spanning the VSV-G protein of the crystal structure (PDB: 5I2S). The hydrophobicity of VSV-G was analyzed using ProtScale (https://web.expasy.org/protscale/). (C) Sequence alignment of envelope proteins in various vesicular stomatitis virus strains. Conserved residues between different vesiculovirus envelope proteins are highlighted in blue. IND, vesicular stomatitis Indiana virus (GenBank: AAA48370.1); NJE, vesicular stomatitis New Jersey virus (NCBI: YP_009047084.1); PIR, Piry virus (Swiss-Prot: Q85213.1); COC, Cocal virus (GenBank: ACB47437.1); CHA, Chandipura virus (NCBI: YP_007641380.1). Alignment of multiple sequences was conducted using DNAMAN version 6 software.

structure.[9,10] Thus, eight sites on VSV-G were chosen for incorporation of hepatocyte-targeting peptides: one site was within the N terminus of VSV-G protein, two sites were within the domain I region, three sites were within the domain III region, and the other two sites were located within the loop region (Figure 3). By site-directed mutagenesis, nine envelope variants with AP peptide incorporation at above eight sites were constructed, respectively (Table S1). Of them, three yielded viable vectors: two with AP peptide insertion at the N terminus of VSV-G, next to the signal peptide with or without a linker (GGGGS), and the other with AP peptide insertion between amino acids 201E and 202G. Similarly, nine envelope variants with CSP peptide insertion were constructed, respectively (Table S1). Among them, four yielded viable vectors: two with CSP insertion at the N terminus of VSV-G next to the signal peptide with or without a linker (GGGGS) and the other one with CSP peptide insertion between amino acids 174K and 175G, the 4th one with CSP peptide insertion between amino acids 351T and 352T. The representative titers of LVs pseudotyped with VSV-G variants are shown in Figures 3D and 3E.

### *In vitro* screening of liver-targeting lentiviral shRNAs against IDOL

The IDOL-targeting siRNAs were designed on the basis of IDOL mRNA sequences from human (RefSeq: NM_013262.4), rhesus monkey (RefSeq: XM_015135555.2), mouse (RefSeq: NM_153789.3), rat (RefSeq: NM_001107344.2), and golden hamster (RefSeq: XM_005066339.3). After alignment, the conserved regions in these IDOL mRNA sequences were analyzed using Designer of

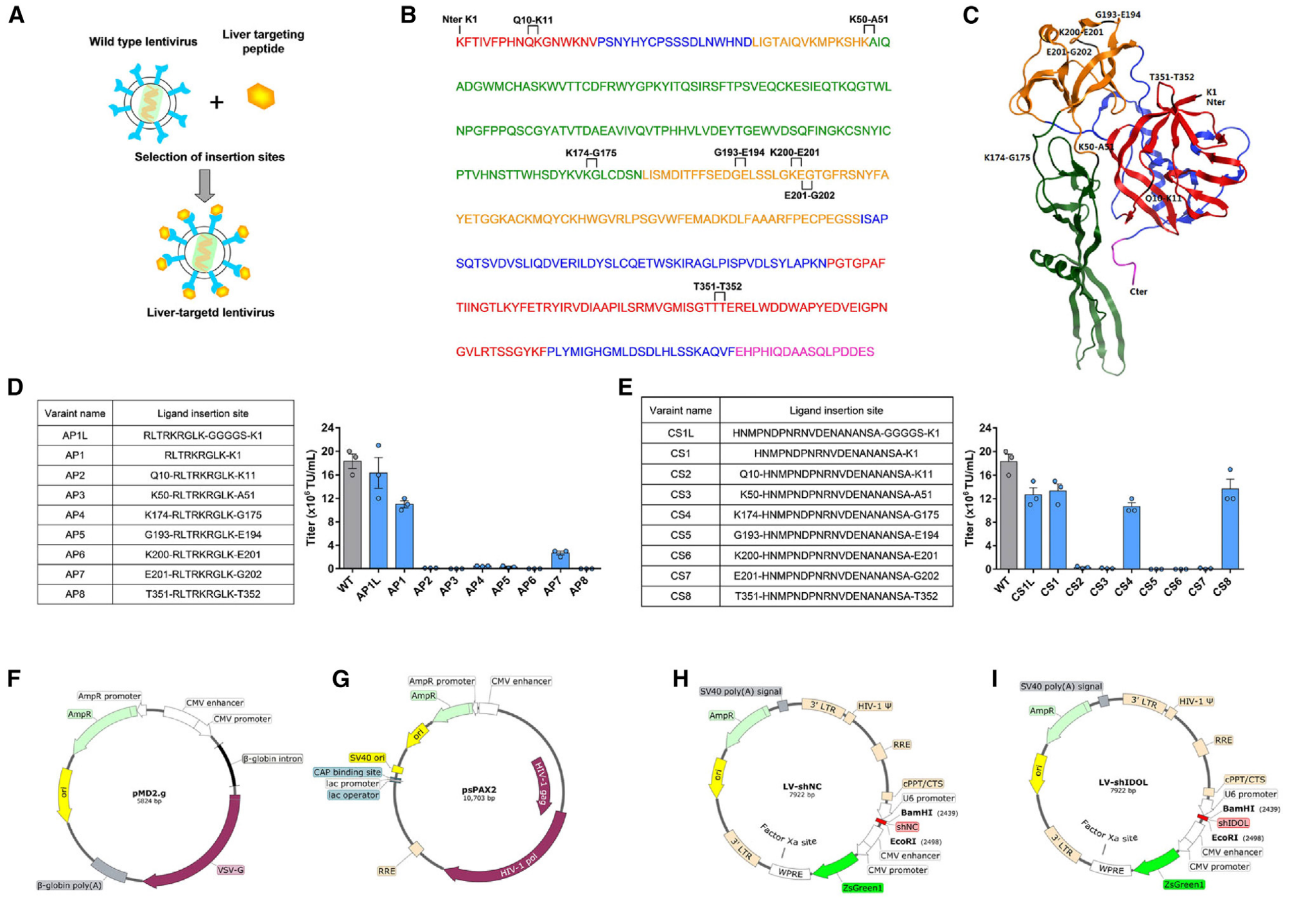


**Figure 3. Liver-targeting peptide insertion sites on VSV-G and representative titers of lentiviral vectors pseudotyped with VSV-G variants**

(A) Scheme of the lentiviral retargeting strategy. Liver-targeting peptide was incorporated into the VSV-G, and the engineered envelope was used to pseudotype lentivirus. (B) Insertion sites of liver-targeting peptides were marked on the amino acid sequence of VSV-G. The domains of VSV-G are displayed in colors: red, lateral domain; blue, trimerization domain; orange, PH domain; green, fusion domain; and magenta, C-terminal part. (C) Crystal structure of the VSV-G protein. Insertion sites of liver-targeting peptides were indicated. The color code for different domains is as the same as in (B). The VSV-G protein crystal structure (PDB: 5I2S) was used and edited in Molecular Operating Environment for Windows. (D and E) VSV-G constructs with ligand insertion sites and representative titers of lentiviral vectors pseudotyped with different VSV-G variants. Error bars represent the standard error of the linear regression used to determine titers. (F and G) Lentiviral plasmids encoding for VSV-G variants (F) and matrix proteins (G). The VSV-G full-length plasmid pMD2.g was used for the construction of variants with peptide insertions. (H and I) Lentiviral shRNA expression plasmids LV-shNC and LV-shIDOL with ZsGreen tag were constructed for lentivirus production with helper plasmid pMD2.g (F) and psPAX2 (G). The shRNA expression vectors were constructed using the U6 pol III promoter on the basis of a 19 nt siRNA sequence (scramble and siIDOL-622) (Table S3). All shRNA sequences have identical loop sequences.

Small Interfering RNA (DSIR) (http://biodev.extra.cea.fr/DSIR/DSIR.html), and three groups of IDOL-targeting siRNAs were generated, which were cross-reactive to human, rhesus monkey, and mouse, to human, rhesus monkey, and rat, and to human, rhesus monkey, and golden hamster targets, respectively. Of them, ten candidate siRNAs (Table S2) were selected on the basis of DSIR-predicted silencing efficacy, the number of potential off-targets, and secondary structure stability calculated using RNAstructure version 6.4 (http://rna.urmc.rochester.edu/RNAstructure.html).

The inhibitory potency of these selected siRNAs on IDOL expression was tested by western blot analysis in human HepG2 and mouse Hepa1–6 cells, respectively. After transfection with 30 nM siRNAs, it was found that siIDOL-619 and siIDOL-622 potently inhibited the expression of IDOL by ~60%, compared with the scramble siRNA group, whereas the others appeared less effective in HepG2 cells (Figures S1A and S1B). Similar results were observed in mouse Hepa1–6 hepatic cells (Figures S2A and S2B).

Also, we detected the regulatory effects of these siRNAs on the function of LDL cholesterol (LDL-C) uptake through suppressing IDOL expression in HepG2 and Hepa1–6 cells. After transfection with 30 nM siIDOL-485, siIDOL-619, siIDOL-622, siIDOL-1406, and siIDOL-1508, DiI-LDL uptake assay was performed. It was observed

that siIDOL-622 most potently increased LDL-C uptake by ∼30% compared with control in HepG2 cells (Figure S1C). Similar results were obtained in mouse Hepa1–6 cells (Figure S2C).

Furthermore, we verified that siIDOL-622 treatment dramatically reduced IDOL levels, resulting in a significant increase in LDLR expression at the cell surface in HepG2 (Figures S1D and S1E) and Hepa1–6 cells (Figures S2D and S2E) by immunofluorescence and flow cytometry analyses. Thus, siIDOL-622 was selected for the subsequent construction of IDOL-shRNA LVs.

The siIDOL-622 sequence and a scrambled siRNA (catalog #A06001NC-RL; Genepharm) beginning with a G were embedded in shRNA scaffolds as previously described.[30] The shRNA oligos contain restriction sites at both ends, a 19 nt sequence that is identical to siRNA sense strand, a hairpin loop sequence, and a 19 nt sequence that is the reverse complement of siRNA sense strand, as well as a poly T termination sequence. The above shRNA sequences are listed in Table S3. The lentiviral expression vectors pLVX-shIDOL and pLVX-shNC were constructed by subcloning the synthetic annealed shRNA duplexes at BamH I/EcoR I sites of pLVX-shRNA2 (catalog #632179; Clontech), allowing shRNA duplexes to be driven by U6 pol III promoter.

Subsequently, the recombinant LVs were pseudotyped with hepatocyte-targeting peptides (AP1L, AP1, AP7, CS1L, CS1, CS4, and CS8) incorporating VSV-G envelopes, respectively (Figures 3D and 3E). The resultant shRNA-expressing LVs were tested for their IDOL silencing efficiency using western blot and qRT-PCR in human HepG2 cell lines, using wild-type VSV-G envelope pseudotyped LV-shIDOL and the scramble siRNA generated LV-shNC as a positive control and a negative control, respectively. As shown in Figures 4A–4C and S7, AP1L-LV-shIDOL, CS1L-LV-shIDOL, and CS8-LV-shIDOL treatment inhibited IDOL expression by ∼60%, leading to a significant up-regulation of LDLR in HepG2 cells, similarly in Hepa1–6 cells (Figures S3A and S3B). In combination with immunofluorescence and flow cytometry analyses, we confirmed that CS8-LV-shIDOL most effectively inhibited the expression of IDOL and elevated cell surface LDLR levels in HepG2 (Figures 4E–4G), Hepa1–6 (Figures S3D–S3F), and the other two cells (Figures S4 and S5). Also, the functional assay indicated that CS8-LV-shIDOL treatment most strikingly enhanced LDL-C uptake by ∼30% both in HepG2 (Figure 4D) and Hepa1–6 cells (Figure S3C), indicating incorporation of hepatocyte-targeting peptide CS8 into VSV-G did not affect the envelope function. Thus, liver-targeted LV CS8-LV-shIDOL was selected for the following *in vivo* studies.

### CS8-LV-shIDOL has extremely high accumulation in liver

To assess the biodistribution of hepatocyte-targeting peptide-incorporated lentiviral shRNA vectors *in vivo*, CS8-LV-shIDOL and LV-shIDOL were administered intravenously via tail vein to mice, respectively. One week after injection, mice were sacrificed, and the fluorescence intensity in five different organs (heart, liver, kidney, lung, and spleen) was detected. As shown in Figure 5, wild-type LV-shIDOL was accumulated mainly in liver and kidney and was less distributed in heart, spleen, and lung, while the liver-targeted LV CS8-LV-shIDOL had extremely high accumulation in liver and nearly undetectable levels in other four organs. The *ex vivo* images of dissected tissues taken by fluorescence microscopy showed similar results (Figure 5C). Thus, a novel liver-targeted lentivirus delivery system has been well established by incorporating the hepatocyte-targeting ligand CS8 peptide into VSV-G protein.

### *In vivo* biosafety of CS8-LV-shIDOL

To test the *in vivo* biosafety of CS8-LV-shIDOL, we assessed routine blood parameters by measuring plasma alanine aminotransferase (ALT), aspartate aminotransferase (AST), alkaline phosphatase (ALP), plasma urea, and creatinine (CREA). Biochemical assay results showed that the blood levels of ALT, AST, ALP, urea, and CREA in mice after injection with CS8-LV-shIDOL for 7 days were not significantly different altered compared with those of the saline-treated group (Figures S6A–S6E), demonstrating that CS8-LV-shIDOL did not show significant toxicity on the liver and kidney. Besides, no tissue or cell damage was observed in heart, liver, spleen, lung, and kidney in mice receiving CS8-LV-shIDOL after histology examination by H&E staining (Figure S6F).

### CS8-LV-shIDOL shows enhanced therapeutic efficacy in lowering serum lipid and alleviating atherosclerotic lesions in mice

The therapeutic effects of liver-targeted LV CS8-LV-shIDOL on hyperlipidemia and atherosclerosis were evaluated in hyperlipidemic mice. Male C57/BL6 mice were fed an atherogenic diet and intraperitoneally injected with poloxamer-407 (P407) at an interval of three days to induce hyperlipidemia and atherosclerosis for 16 weeks.[31–33] Meanwhile, mice received $1 \times 10^8$ TU LV-shNC (negative control), LV-shIDOL, and CS8-LV-shIDOL per animal intravenously via the tail vein every eight weeks. Twenty-four hours after final injection of P407, mice were fasted for 8 h and then euthanized for blood and tissue collection. By analysis on the serum lipids, it was shown that the LV-shIDOL and CS8-LV-shIDOL groups had 29.74% and 45.82% decreases in serum LDL-C, as well as 14.98% and 30.05% decreases in serum total cholesterol (TC), respectively compared with the LV-shNC group (Figures 6B and 6C). The serum LDL-C and TC levels in the liver-targeted CS8-LV-shIDOL group were significantly lower than those in non-targeted LV-shIDOL group, indicating that incorporation of the hepatocyte-targeting ligand CS8 peptide into VSV-G protein facilitated liver-specific cellular uptake of LV-shIDOL and led to enhanced IDOL silencing efficiency. Furthermore, atherosclerotic lesion development of aortic root was measured using oil red O staining, it was found that atherosclerotic plaques in the CS8-LV-shIDOL-treated group were significantly milder than those in LV-shIDOL group and much less severe than those in the saline- and LV-shNC-treated groups (Figure 6D). Although both LV-shIDOL and CS8-LV-shIDOL remarkably inhibited the expression of hepatic IDOL, CS8-LV-shIDOL showed an increase of ∼60% in IDOL silencing efficiency, leading to higher potency in up-regulating LDLR, compared with non-targeted LV-shIDOL (Figures 6E–6G).

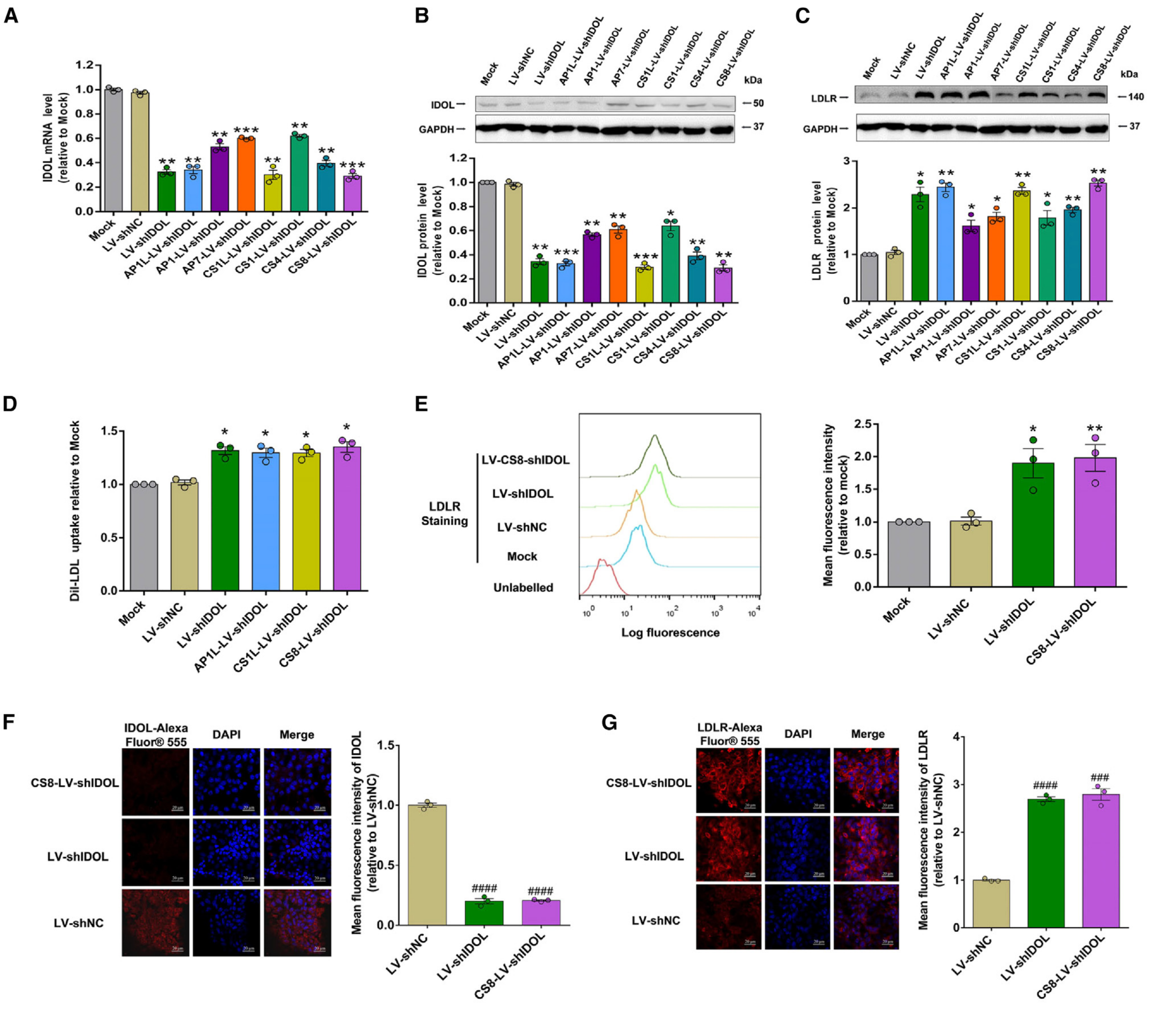


**Figure 4. Functional analysis on the liver-targeted lentiviral shRNAs against IDOL *in vitro***

(A–D) HepG2 cells were transfected with LV-shNC (negative control), LV-shIDOL (lentiviral IDOL-shRNA with wild-type VSV-G), and lentiviral IDOL-shRNAs with different VSV-G variants, respectively. After 48 h, the levels of IDOL mRNA in HepG2 cells were quantified using qRT-PCR (A). After 72 h, the levels of IDOL and LDLR protein were measured using western blot (B and C), DiI-LDL uptake levels were measured using a multimode reader (D), then normalized to mock control. (E–G) Effects of CS8-LV-shIDOL on IDOL and LDLR expression in HepG2 cells. After transfection with LV-shNC, LV-shIDOL, and CS8-LV-shIDOL for 72h, IDOL protein levels were visualized using immunofluorescence assay (F). The cell surface LDLR protein levels were determined using flow cytometry (E) and evaluated using immunofluorescence assay (G). $^{*}p < 0.05$, $^{**}p < 0.01$, and $^{***}p < 0.001$ vs. mock control; $^{\#\#\#}p < 0.001$ and $^{\#\#\#\#}p < 0.0001$ vs. LV-shNC control (unpaired Student's t test). Results are given as mean ± SEM of three independent experiments.

This was further confirmed by immunofluorescence analysis (Figure 6H). Taken together, liver-targeted CS8-LV-shIDOL had higher efficiency in hepatotropic delivery of IDOL-shRNA than non-targeted LV-shIDOL, leading to enhanced potency in inhibiting IDOL, up-regulation of LDLR, reduced serum LDL-C and TC, and improved therapeutic efficacy in reducing atherosclerotic lesions in hyperlipidemic mice.

### CS8-LV-shIDOL exhibits improved therapeutic effects in reducing hepatic lipid accumulation and attenuating liver injury in mice

To assess the therapeutic efficacy of CS8-LV-shIDOL on reducing hepatic fat accumulation and attenuating liver injury in mice, histological analyses on liver sections using oil red O and H&E staining were performed. As shown in Figure 7A, CS8-LV-shIDOL treatment

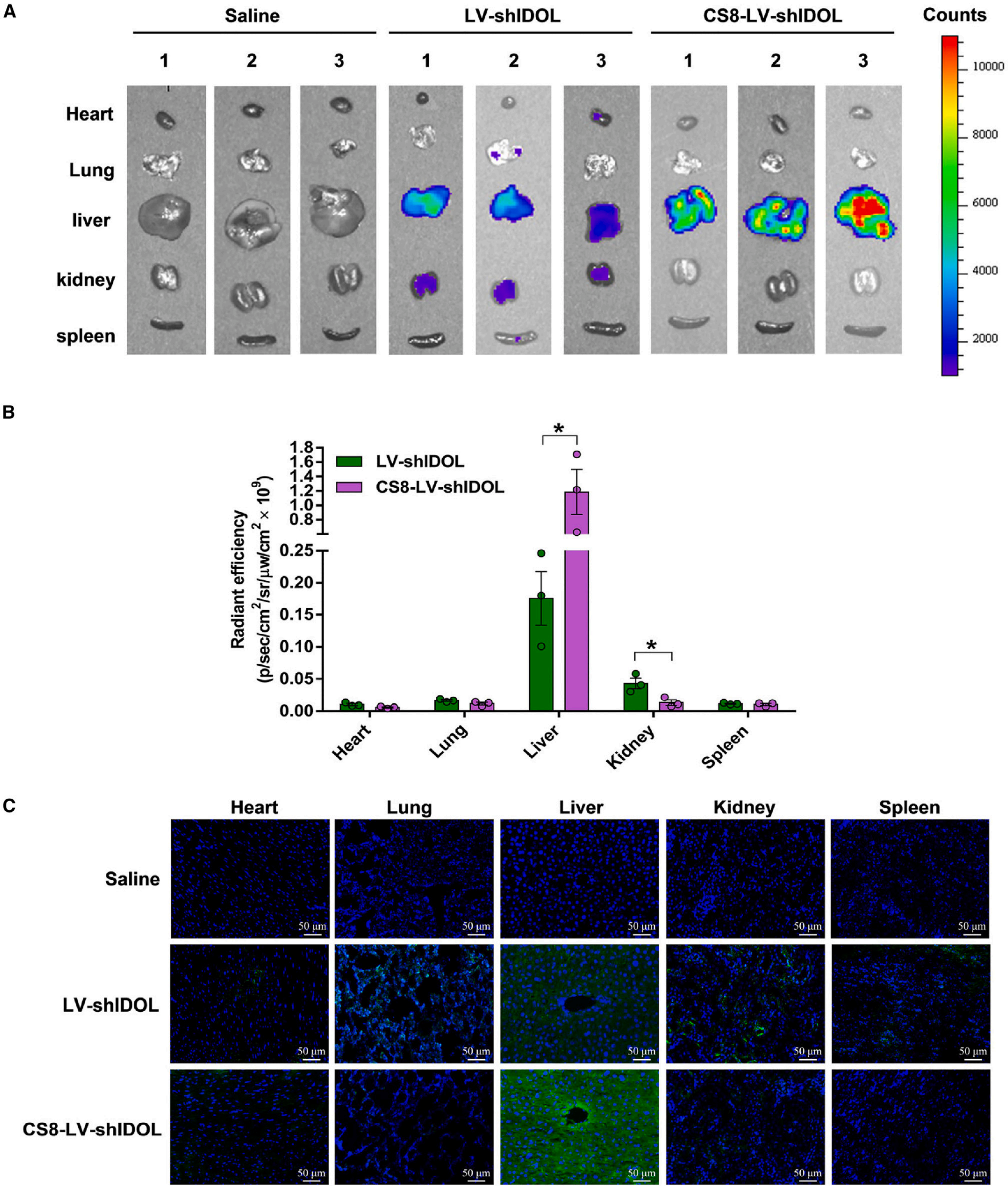


(legend on next page)

reduced hepatic lipid deposition more potently than LV-shIDOL treatment, although both notably decreased lipid deposition compared with the shNC treatment group. Additionally, CS8-LV-shIDOL treatment alleviated liver injury more effectively than in the LV-shIDOL group (Figure 7B). Furthermore, it was confirmed that administration of liver-targeted CS8-LV-shIDOL caused an average 42.3% decrease in hepatic triglyceride (TG) content, which appeared to be lower than the 28.9% reduction attained with non-targeted LV LV-shIDOL (Figure 7B). Taken together, these data demonstrated that liver-targeted CS8-LV-shIDOL was much more efficacious than non-targeted LV-shIDOL in reducing hepatic lipid deposition and ameliorating liver injury in mice.

### LRP mediates the targeted entry of CS8-LV-shIDOL into hepatocytes

To identify the receptor that mediates the targeted entry of CS8-LV-shIDOL into hepatocytes, HepG2 cells were incubated with anti-LDLR monoclonal antibody at 37°C for 1 h to mask cell surface LDLR, a natural entry receptor for vesicular stomatitis virus,[34] which is widely expressed in various tissues, such as liver, spleen, intestine, adrenals, neurons, microglia, and white and brown adipose tissues.[21,22] Then, LV-shNC, LV-shIDOL, and CS8-LV-shIDOL were added. After incubation at 37°C for 30 min, the supernatant containing LV was replaced with fresh MEM medium and incubated at 37°C for additional 16 h. Subsequently, the plates were rinsed with PBS and the infected HepG2 cells were analyzed for ZsGreen expression. It was observed that wild-type LV infection was efficiently blocked by anti-LDLR monoclonal antibody, while CS8-LV-shIDOL displaying CS8-targeting ligand retained infectivity (Figures 8A–8C). However, when HepG2 cells were treated with monoclonal antibodies against both LDLR and low-density lipoprotein receptor-related protein (LRP) infection of HepG2 cells by CS8-LV-shIDOL was completely blocked (Figures 8A–8C). Thus, it was demonstrated that the targeted entry of CS8-LV-shIDOL into hepatocytes was mediated by the interaction of CS8 ligand with the LRP.

### *In silico* docking of CS8-LV-shIDOL with LRP

The ligand-receptor interaction details for the affinity of CSP peptide to LRP were investigated using computer-based homology modeling and molecular docking analyses. First, the three-dimensional (3D) structure of CS8 peptide-incorporated VSV-G (GenBank: OQ561788) was built on the basis of the wild-type VSV-G crystal structure (PDB: 5I2S) (Figure 8D) using I-TASSER (http://zhanglab.ccmb.med.umich.edu/I-TASSER/). As shown in Figure 8E, it was observed that the targeting ligand CSP peptide was properly displayed without disturbing the native structure of VSV-G, implying the VSV-G engineered CS8-LV-shIDOL might retain a high level of vector titer, enabling it to favorably interact with the target receptor LRP. Afterward, the modeled structure of CS8 peptide-incorporated VSV-G was docked with the crystal structure of LRP1 CR.7 (PDB: 1J8E) using HawkDock web server (http://cadd.zju.edu.cn/hawkdock/), and the binding free energy ($\Delta G_{bind}$) of the ten top-ranked binding poses was calculated in HawkDock web server using the molecular mechanics/GB surface area (MM/GBSA) method. On the basis of the results of docking, the docked pose with the lowest binding free energy was selected as the best conformation to visualize the key interacting residues in the docked complex using Molecular Operating Environment for Windows (Chemical Computing Group Inc., Montreal, QC, Canada). As indicated in Figure 8F, it was revealed that the interaction residues in CS8 peptide-incorporated VSV-G including Asn9 and Lys 11 (in VSV-G), Asn359, and Arg360 (in CS8 peptide) form four hydrogen bonds against Asn13, Ser14, Asp32, and Thr37 in LRP1 CR.7.

## DISCUSSION

In the field of gene therapy, therapeutic viruses have provided treatment options for diseases that are beyond traditional approaches.[35] Compared with other virus vector systems, LVs hold a unique set of advantages, including low immune response, high cargo capacity, and efficient transduction of nondividing cells.[7,36,37] To date, two lentivirus-based *ex vivo* gene therapies, Kymriah (Novartis) and Zynteglo (bluebird bio), have been approved.[3] In addition, direct *in vivo* clinical applications of lentivirus have been reported for the treatment of Parkinson's disease and AMD.[4,5] However, the broad tropism of VSV-G pseudotyped lentivirus makes it a non-ideal gene therapy vector,[38] thus it is required to augment its infection specificity to the particular target cells in order to enhance the therapeutic efficacy and minimize the systemic toxicity. In this work, we developed a novel liver-targeted lentivirus-based system for targeted delivery of IDOL-shRNA into the liver with an improved cholesterol-lowering efficacy.

Since the crystal structure of the prefusion form of VSV-G has been previously delineated,[10] it appears to be feasible to identify the potential sites in VSV-G that are exposed on the molecule surface and tolerant of the insertion of targeting ligands via computational analysis. Theoretically, flexible loop regions with unconserved sequences and rich in hydrophilic amino acids are preferred because of their favorable accessibility to the protein surface.[28,29] Accordingly, eight potential sites which could tolerate an insertion of 20-amino-acid-long peptide were identified in VSV-G. Subsequently, two liver-targeted peptides were incorporated into VSV-G at these potential insertion sites, respectively. By analysis on the titers of LVs pseudotyped with VSV-G variants, 7 VSV-G variants with titers comparable with the parent were selected for subsequent IDOL-shRNA LV construction.

**Figure 5. Biodistribution of CS8-LV-shIDOL in C57/BL6 mice**

(A and B) Liver-targeted lentiviral vector CS8-LV-shIDOL was injected into the tail veins of C57/BL6 mice at a dose of $1 \times 10^8$ TU per mouse. Control mice were injected with an equivalent dose of wild-type lentiviral vector LV-shIDOL or saline. One week after injection, mice were sacrificed, and the heart, kidney, liver, lung, and spleen were taken for imaging *ex vivo*. Fluorescence in collected organs was measured and quantified using the IVIS Imaging System. (C) Tissues were incubated at 4°C in 4% paraformaldehyde for 48 h. Then, sections were stained using DAPI prior to viewing with a fluorescence microscope.

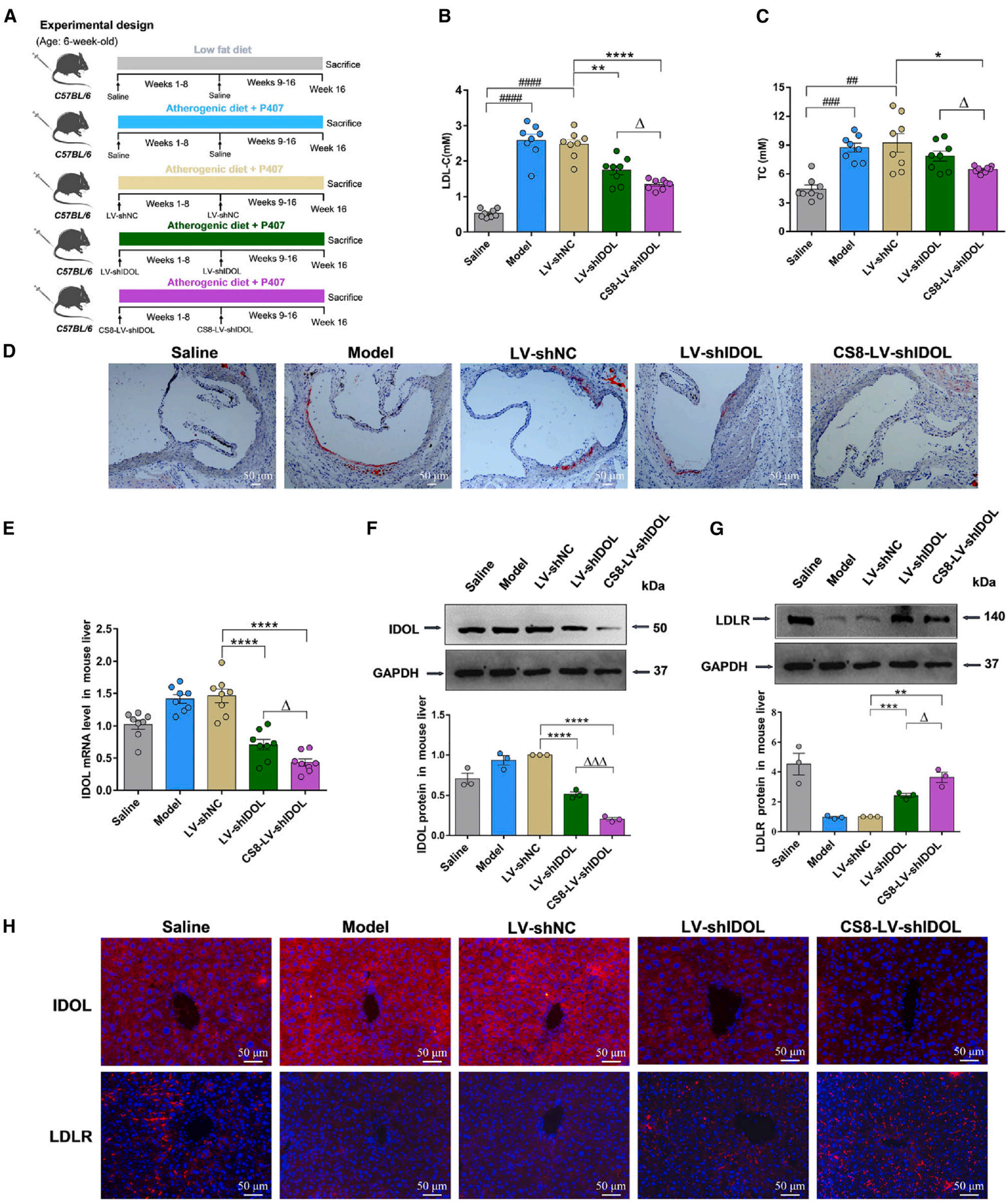


(legend on next page)

IDOL, as an E3-ubiquitin ligase promoting the ubiquitination and degradation of hepatic LDLR, appears to be a promising therapeutic target for treating dyslipidemia and ASCVD.[20–22] To inhibit hepatic IDOL expression in this work, various IDOL-targeting siRNAs were designed on the basis of the homologous regions in IDOL mRNA sequence across human, rhesus monkey, mouse, rat, and golden hamster. By *in vitro* assay, siIDOL-622 was screened out with the most potent silencing effect on IDOL expression, the strongest regulatory function in up-regulating LDLR and increasing LDL-C uptake in HepG2 and Hepa1–6 cells.

In order to facilitate liver-targeted delivery of therapeutic siRNA against IDOL to hepatocytes after systemic delivery, on the basis of siIDOL-622 sequence, we constructed recombinant lentivirus IDOL-shRNAs pseudotyped with various liver-targeting ligand-modified VSV-G envelopes. After *in vitro* assay, it was found that CS8-LV-shIDOL had the highest potency in repressing IDOL expression, up-regulating LDLR, and elevating LDL-C uptake in HepG2 and Hepa1–6 cells, implying that incorporation of hepatocyte-targeting peptide CS8 into VSV-G did not disturb the envelope function.

Furthermore, we investigated the targeted delivery of CS8-LV-shIDOL to hepatocytes *in vivo*. It was observed that wild-type LV LV-shIDOL was distributed mainly in liver and kidney and less distributed in heart, spleen, and lung, while the liver-targeted LV CS8-LV-shIDOL had extremely high accumulation in liver and nearly undetectable levels in other four organs, indicating that incorporating the hepatocyte-targeting ligand CS8 peptide into VSV-G protein greatly improved the liver-targeted delivery of IDOL-shRNA to liver. Besides, we evaluated the *in vivo* therapeutic effects of CS8-LV-shIDOL on hypercholesterolemia and atherosclerosis in P407-induced hyperlipidemic mice fed an atherogenic diet. The data indicated that CS8-LV-shIDOL had much enhanced therapeutic efficacy in reducing serum lipid and alleviating atherosclerotic lesions in mice compared with wild-type LV-shIDOL group. Beyond that, CS8-LV-shIDOL treatment showed superior therapeutic effects in attenuating hepatic lipid accumulation and alleviating liver injury over wild-type LV-shIDOL in mice. However, how IDOL inhibition is linked to the reduction of hepatic lipid and the attenuation of liver injury remains to be addressed.

Additionally, we explored the mechanism by which the hepatocyte-targeting CS8 peptide promoted the liver-targeted delivery of CS8-LV-shIDOL to liver. It has been known that wide-type VSV-G pseudotyped lentivirus enters cells through LDLR, a highly ubiquitous receptor, which leads to its broad tropism.[34,39,40] Interestingly, the CSP of *P. falciparum* is involved in sporozoite entry into hepatocytes,[11,12] and interacts with LRP in liver cells.[41] As an LDLR family member, LRP is expressed at functionally significant levels in hepatocytes and LRP-mediated endocytosis of apoE-enriched chylomicron remnants occurs largely in liver.[42,43] Also, a peptide of T7 phage protein has been revealed to mediate the targeting of hepatocytes via LRP.[44] Thus, we speculated that CS8-LV-shIDOL can target liver cells via LRP. To test this speculation, an LDLR antibody was used to mask the LDLR in HepG2 cells prior to infection, thus allowing the wild-type LV-shIDOL infection was blocked. As a result, the envelope-engineered CS8-LV-shIDOL showed much higher infection levels relative to wild-type LV-shIDOL. Conversely, when the cells were treated with monoclonal antibodies against both LDLR and LRP, CS8-LV-shPCSK9 infection was completely blocked. Thus, we demonstrated that LRP mediates the targeted entry of CS8-LV-shIDOL into hepatocytes. Moreover, we uncovered the ligand-receptor interaction details for the affinity of CS8 peptide-incorporated VSV-G to LRP using computer-based homology modeling and *in silico* docking analysis.

In summary, we successfully developed a novel liver-targeted lentivirus vector system by incorporating a hepatocyte-targeted peptide derived from CSP into the lentivirus envelope for liver-targeted delivery of IDOL-shRNA to specifically inhibit hepatic IDOL expression, thus enabling more potent reduction of hypercholesterolemia and alleviating atherosclerosis. This hepatocyte-targeted lentivirus delivery system has great potential for liver-targeted transgene therapy.

## MATERIALS AND METHODS

### Materials

MEM (catalog #41500034) and Opti-MEM (catalog #31985070) were purchased from Thermo Fisher Scientific (Waltham, MA). Fetal bovine serum (FBS) (catalog #F2442), penicillin G sodium salt (catalog #PENNA), and streptomycin solution (catalog #5711) were obtained from Millipore Sigma (Burlington, MA). Lipofectamine 3000 reagent (catalog #L3000015) was purchased from Invitrogen (Carlsbad, CA). Linear polyethyleneimine (LPEI; 25 kDa) (catalog #23966) was purchased from Polysciences (Warrington, PA). RIPA lysis buffer (catalog #R0020) was obtained from Solarbio (Beijing, China). Phenylmethyl sulfonyl fluoride (PMSF; catalog #M145-5G) was purchased from Amresco (Solon, OH). P407 (Pluronic F-127; catalog #P2443) was obtained from Sigma-Aldrich (Parsippany, NJ). Rabbit anti-LDLR antibody (catalog #ab52818; RRID: AB_881213) was obtained from Abcam (Cambridge, UK). Rabbit anti-IDOL antibody (catalog #D126668), rabbit anti-GAPDH

**Figure 6. Therapeutic effects of CS8-LV-shIDOL in mice**

(A) Schematic diagram of the experimental procedure to develop an atherosclerosis model in C57/BL6 mice. Solid arrows indicate the time points for injection of lentiviral vectors. (B and C) P407-induced hyperlipidemic mice fed an atherogenic diet were injected with LV-shNC, LV-shIDOL, and CS8-LV-shIDOL ($1 \times 10^8$ TU per mouse at intervals of 8 weeks) for 16 weeks. At the end of experiment, serum, aorta, and liver samples were collected. CS8-LV-shIDOL was more effective in lowering serum LDL-C and TC compared with LV-shIDOL. Results are expressed as mean ± SEM (n = 8 per group). (D) Atherosclerotic plaques in aortic root were measured using oil red O staining. (E–H) The hepatic IDOL and LDLR levels in LV-shIDOL and CS8-LV-shIDOL-treated groups were detected using qRT-PCR (E), western blot (F and G) and immunofluorescence assay (H). *$p < 0.05$, **$p < 0.01$, and ****$p < 0.0001$ vs. LV-shNC group. $^{\#\#}p < 0.01$, $^{\#\#\#}p < 0.001$, and $^{\#\#\#\#}p < 0.0001$ vs. saline group. $^{\Delta}p < 0.05$ and $^{\Delta\Delta\Delta}p < 0.001$ vs. LV-shIDOL group. Scale bar, 50 μm. Data are representative of 3 independent experiments with similar results.

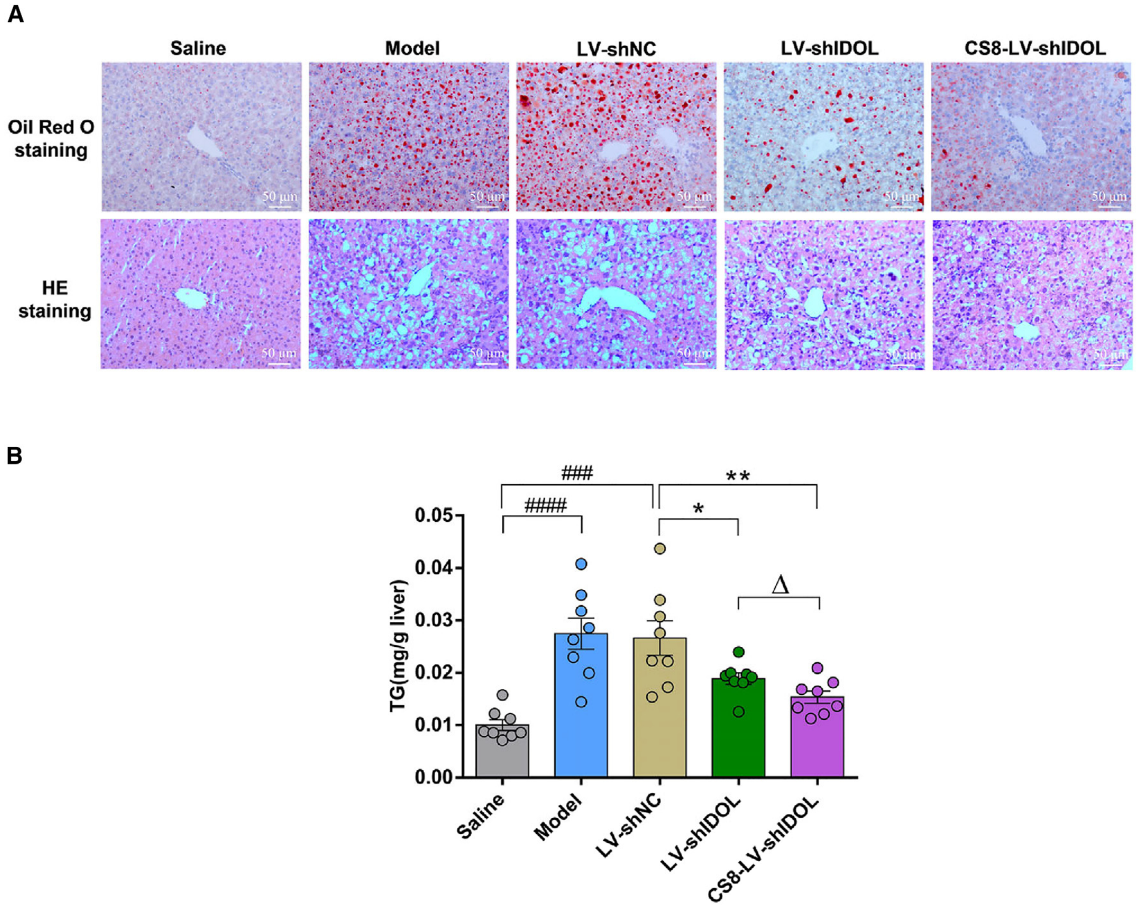


**Figure 7. Effects of CS8-LV-shIDOL on hepatic lipid accumulation in mice**

(A) P407-induced hyperlipidemic mice fed an atherogenic diet were injected with LV-shNC, LV-shIDOL, and CS8-LV-shIDOL ($1 \times 10^8$ TU per mouse at intervals of 8 weeks) for 16 weeks. At the end of experiment, liver samples were collected. Liver sections were stained with oil red O (top panel) and H&E (bottom panel). (B) Effects of CS8-LV-shIDOL on hepatic TG contents. $^*p < 0.05$ and $^{**}p < 0.01$ vs. LV-shNC group; $^{###}p < 0.001$ and $^{####}p < 0.0001$ vs. saline group; $^{\Delta}p < 0.05$ and $^{\Delta\Delta}p < 0.01$ vs. LV-shIDOL group. Scale bar, 50 μm. Data are representative of 3 independent experiments with similar results.

antibody (catalog #D110016), rabbit anti-β-actin antibody (catalog #D110001), Alexa Fluor 488-conjugated goat anti-rabbit IgG (catalog #D110061), and Alexa Fluor 555-conjugated goat anti-rabbit IgG (catalog #D110070) were purchased from BBI (Toronto, ON, Canada). BeyoFast SYBR Green qPCR Mix (catalog #D7262-5ml), BeyoRT II First Strand cDNA Synthesis Kit with gDNA Eraser (catalog #D7170M), and rabbit anti-LRP1 antibody (catalog #AF1000) were obtained from Beyotime (Shanghai, China). Horseradish peroxidase (HRP)-conjugated goat anti-rabbit Ig G (H + L) (catalog #FMS-Rb01) was purchased from Fcmacs Biological (Nanjing, China). LDL labeled with 1,1′-dioctadecyl-3,3,3′,3′-tetramethyl-indocarbo-cyanine perchlorate (DiI-LDL) was obtained from Yiyuan Biotechnologies (catalog #YB-0011; Guangzhou, China). Assay kits for LDL-C (catalog #A113-1), TC (catalog #A111-1), TG (catalog #A110-1), ALP (catalog #A059-2), ALT (catalog #C009-2), AST (catalog #C010-2), CREA (catalog #C011-2), and blood urea nitrogen (BUN) (catalog #C013-2) were obtained from Nanjing Jiancheng Bioengineering Institute (Nanjing, China).

### Construction of lentivirus envelope mutant plasmids

The VSV-G expression plasmid pMD2.g was used as the template for all mutant constructions. Sites for mutagenesis were chosen on the basis of a VSV-G crystal structure,[9,10] which provided the means for the preliminary structural identification of surface loop regions that were envisioned as possibly tolerating foreign peptide sequence insertions. Liver cell-specific peptides derived from AP (RLTRKRGLK)[14] and *P. falciparum* circumsporozoite (CS) protein (HNMPNDPNRNVDENANANSAYC)[13] were inserted into appropriate places in VSV-G protein by overlapping PCR. The peptide sequences and insertion sites on VSV-G are listed in Table S1. Insertion mutation of liver-targeting peptides in VSV-G encoded in pMD2.G (Addgene, Cambridge, MA) was carried out using two sets of primers for every insertion. The primers used are listed in Table S4. The resulting PCR products and the plasmid pMD2.G were digested with restriction enzymes and ligated to obtain the modified VSV-G constructs.

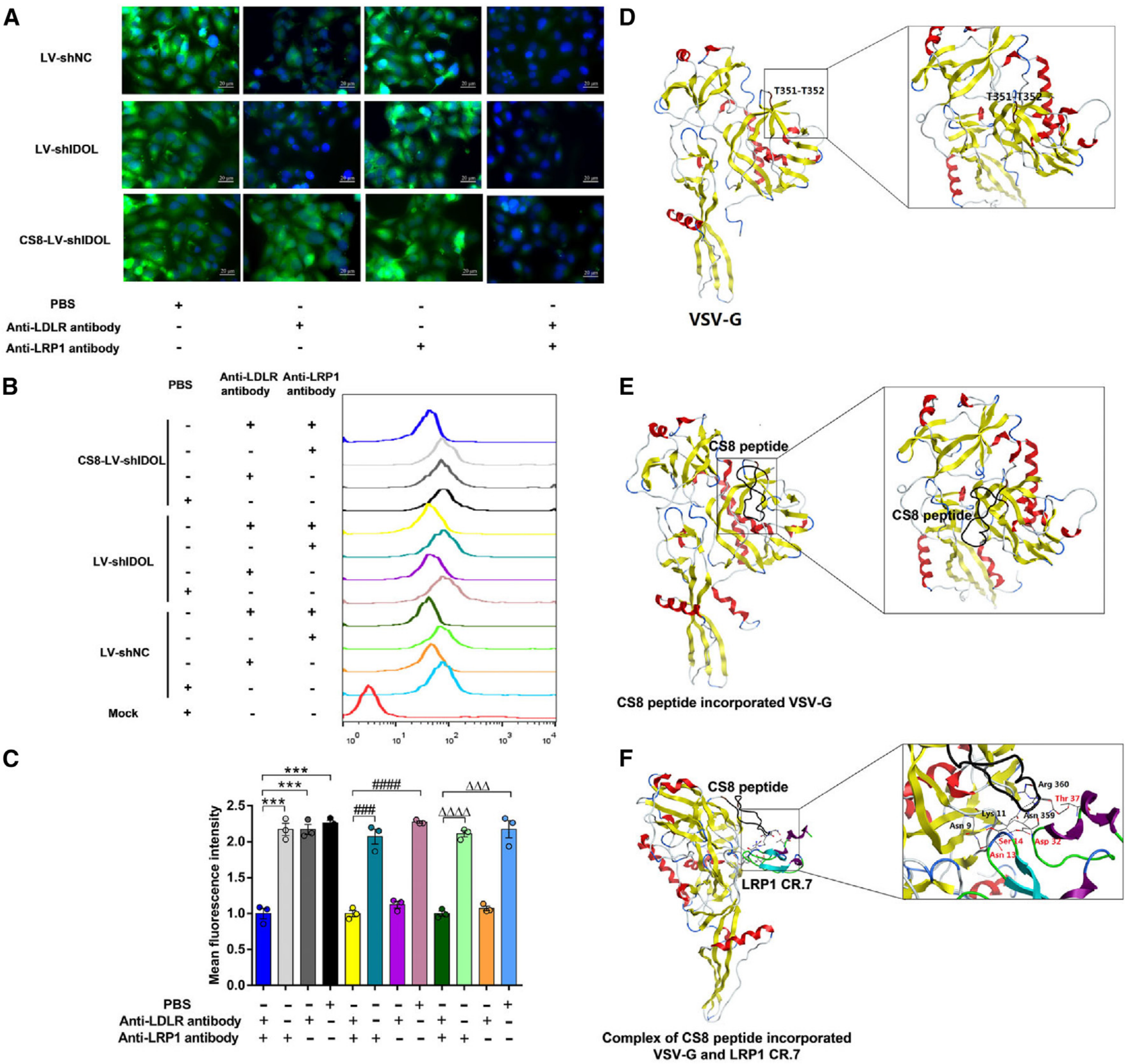


**Figure 8. Binding of CS8 peptide-incorporated VSV-G to LRP mediates the targeted entry of CS8-LV-shIDOL into hepatocytes**

(A and B) HepG2 cells were treated with anti-LDLR and/or anti-LRP1 monoclonal antibody for 1 h before the addition of lentiviral vectors. After transfection with LV-shNC, LV-shIDOL, and CS8-LV-shIDOL for 1 h, cells were washed and incubated at 37°C for 16 h. Fluorescence was detected using microscopy (A) and flow cytometry (B and C). $^{***}p < 0.001$; $^{\#\#\#}p < 0.001$ and $^{\#\#\#\#}p < 0.0001$; and $^{\Delta\Delta\Delta}p < 0.001$ and $^{\Delta\Delta\Delta\Delta}p < 0.0001$ (unpaired Student's t test). Results are given as mean ± SEM of three independent experiments. (D) The 3D structure of VSV-G protein (PDB: 5I2S). (E) Modeling of CS8 peptide-incorporated VSV-G (CS8-VSV-G) was constructed using I-TASSER, and CS8 peptide is shown in black. All the structures were edited using Molecular Operating Environment for Windows. (F) *In silico* docking of CS8 peptide-incorporated VSV-G with LRP1 CR.7 using HawkDock. Key residues involved in the interactions are represented as stick models and indicated in black font for CS8-VSV-G and in red font for LRP1 CR.7. The hydrogen bonds are shown as black dotted lines.

### Design of siRNAs targeting IDOL and construction of lentiviral shRNA vectors

The mRNA sequence of IDOL can be retrieved using RefSeq in the National Center for Biotechnology Information (NCBI) Entrez Gene database, and DNAMAN (version 6) (Lynnon Biosoft) was used to search for mRNA conserved sequences. The IDOL-targeting siRNAs were designed using DSIR version 7 (http://biodev.extra.cea.Fr/DSIR/DSIR.html)[45] and RNAstructure version 6.4 (http://rna.urmc.rochester.edu/RNAstructure.html).[46] Moreover, the homology analysis of the candidate siRNAs was conducted by using BLAST provided from NCBI GeneBank with the transcript reference sequence database (Transcript Reference Sequences) for excluding the possibility that siRNA non-specifically inhibits genes other than IDOL. All siRNAs (Table S2) were synthesized and purified by GenePharma (Shanghai, China) as well as dissolved to a concentration of 20 μM with sterilized and RNase-free water according to the manufacturer's protocol. The selected IDOL-targeting siRNA and a scrambled siRNA (catalog #A06001NC-RL; Genepharm) were embedded in shRNA scaffolds as previously described,[30] and the shRNA sequences used in this study are listed in Table S3. The lentiviral expression vectors

pLVX-shIDOL and pLVX-shNC were constructed by subcloning the synthetic annealed shRNA duplexes at BamH I/EcoR I sites of pLVX-shRNA2 (catalog #632179; Clontech).

#### Production of recombinant LVs with modified envelopes

LVs were produced as previously described[47] with slight modification. Briefly, lentiviral plasmids encoding for VSV-G variants, matrix proteins (psPAX2), and ZsGreen (pLVX-shRNA2) were used to transfect 293T cells by 25 kDa LPEI. Forty-eight and 72 h post-transfection, lentivirus containing supernatant was harvested and concentrated using a precipitation-based method using PEG8000. Twenty-five percent polyethylene glycol (PEG) 8000 and 0.75 M NaCl were added to LV containing supernatant and incubated at 4°C for 5 h, with mixing every 20 min. Subsequently, the mixture was pelleted at 7,000 × *g* for 15 min, and the pellet was resuspended in PBS. The final concentrated lentivirus was titered immediately and then aliquoted and stored at −80°C for further analysis. Viral titers were determined as described previously.[48,49] The titer of viral preparations with different VSV-G variants was measured by performing transduction serial dilutions of concentrated lentivirus in 96-well plates with 293T cells seeded at 30,000 cells per well in presence of 8 mg/mL polybrene. The fraction of transfected cells (ZsGreen) was determined by counting fluorescent versus nonfluorescent cells using a Zeiss AX10 fluorescence microscope. The titer was calculated on the basis of the following formula: $\text{TU ml}^{-1} = (F \times N \times D \times 1{,}000)/V$, where $F$ is the percentage of fluorescent cells (ZsGreen), $N$ is the number of cells at the time of transduction (corresponding to about $1 \times 10^{-5}$ cells per well), $D$ is the fold dilution of vector sample used for transduction, and $V$ is the volume of diluted vector sample added into each well for transduction.

#### Cell culture and treatments

Human hepatoma (HepG2), mouse hepatoma (Hepa1–6), and HEK293T cells were obtained from China Infrastructure of Cell Line Resources (Beijing, China) and maintained in MEM (HepG2 cells) or DMEM (Hepa1–6 and HEK293T cells) supplemented with 10% FBS, 100 U/mL penicillin, and 100 μg/mL streptomycin in a humidified 5% $CO_2$ atmosphere at 37°C. Mouse normal cell line AML12 was obtained from the Cell Bank of the Chinese Academy of Science (Shanghai, China) and maintained in DMEM/F12 supplemented with 10% FBS, 10 μg/mL insulin, 5.5 μg/mL transferrin, 5 ng/mL selenium, 40 ng/mL dexamethasone, 100 U/mL penicillin, and 100 μg/mL streptomycin in a humidified 5% $CO_2$ atmosphere at 37°C. For LDLR protein quantification and DiI-LDL uptake measurement experiments, Opti-MEM medium was used instead of medium containing 10% FBS after transfection for 48 h.

For siRNA transfection, about $5 \times 10^5$ HepG2 or Hepa1–6 cells were seeded per well into a 6-well plate and transfected with 30 nM negative control or 30 nM specific IDOL siRNAs (Table S2) using Lipofectamine 3000 (Invitrogen), respectively. After transfection for 48 h, cells were rinsed with PBS and incubated with serum-free Opti-MEM at 37°C for 24 h. IDOL, LDLR expression levels, and LDL uptake function were detected using western blot, immunofluorescence, flow cytometry analysis, and DiI-LDL staining, respectively. Quantification of LDLR protein levels was performed using western blot analysis.

For lentivirus shRNA transduction, about $5 \times 10^5$ HepG2, Hepa1–6, or AML12 cells were seeded per well into a 6-well plate and transduced with $5 \times 10^6$ TU lentivirus in the presence of 4 μg/mL polybrene (catalog #H9268; Sigma-Aldrich). After transduction for 24 h, the virus-containing medium was discarded and replaced with fresh growth medium. The cells were then cultured for additional 24 h and then rinsed with PBS and incubated with serum-free Opti-MEM at 37°C for 24 h. IDOL, LDLR expression levels, and LDL uptake function were detected using western blot, immunofluorescence, flow cytometry analysis, and DiI-LDL staining, respectively. Quantification of LDLR protein levels was performed using western blot analysis.

#### Western blot

Western blot was performed to measure the protein expression levels in HepG2 and Hepa1–6 cells or tissues, as previously described.[50] Western blot analysis was performed to measure the protein expression levels in cells. HepG2 and Hepa1–6 cells were washed three times in ice-cold PBS and then lysed in ice-cold RIPA lysis buffer (50 mM Tris, 150 mM Na Cl, 1 mM ethylenediaminetetraacetic acid [EDTA], 1% Triton X-100, 0.5% sodium deoxycholate, and 0.1% SDS, [pH 7.4]) containing 1 mM PMSF. After centrifugation at 12,000 × *g* for 15 min at 4°C, the supernatant was harvested. Total soluble protein was measured using BCA protein assay kit (Biouniquer, Beijing, China). An equal amount of protein from each sample was loaded in each lane of a 10% SDS-PAGE gel and separated. The separated proteins were electrophoretically transferred onto a 0.22 μm PVDF membrane (Merck Millipore, Darmstadt, Germany). The membrane was blocked with a solution of 0.1% (vol/vol) TBS-Tween 20 (TBST) containing 5% (w/v) nonfat milk for 1 h at room temperature, then incubated with corresponding primary antibodies against IDOL (1:1,000), LDLR (1:2,000), GAPDH (1:1,000), β-actin (1:1,000), and LRP1 (1:1,000) at 4°C overnight, followed by incubation with appropriate HRP-conjugated secondary antibodies (1:5,000, Fcmacs Biological) for 1–2 h at room temperature. After washing three times with TBST for 5 min, protein bands were developed using ECL (Thermo Fisher Scientific) and quantified using ImageJ software (National Institutes of Health, Bethesda, MD).

#### qRT-PCR analysis

Total RNA was extracted from HepG2 and AML12 cells and liver tissues by using RNAiso Plus reagent according to the manufacturer's instructions and quantified by measuring A260nm using Thermo NanoDrop 2000 (Thermo Fisher Scientific). For mRNA quantification, cDNA was generated using the BeyoRT II First Strand cDNA Synthesis Kit with gDNA Eraser. The qRT-PCR analyses were performed using BeyoFast SYBR Green qPCR Mix on a MX3000PTM qRT-PCR instrument (Agilent Technologies, Santa Clara, CA). To measure mRNA expression levels, the data were normalized to the housekeeping gene β-actin or GAPDH. Primers for each gene are

listed in Table S5. The $2^{-\Delta\Delta Ct}$ method[51] was used to calculate relative gene expression levels.

### LDL uptake assay

The assay was performed as described previously[52] with slight modification. Briefly, HepG2 and Hepa1–6 cells were maintained in MEM and DMEM supplemented with 10% FBS, respectively. The cells were seeded in 96-well black plates at a density of $1 \times 10^4$ cells per well and grown to 70%–80% confluence. Following transfection or transduction for 48 h, HepG2 and Hepa1–6 cells were incubated with serum-free Opti-MEM for another 24 h, and 20 μg/mL DiI-LDL was added and incubated in the dark for additional 4 h. Cells incubated with Opti-MEM without DiI-LDL and cells incubated with Opti-MEM in the presence of 20 μg/mL DiI-LDL were used as negative control and control for normalization, respectively. After rinsing 3 times with PBS, LDL uptake was measured using a fluorescence plate reader (Varioskan Fflash; Thermo Fisher Scientific) at an excitation wavelength of 520 nm and an emission wavelength of 580 nm.

### Flow cytometric analysis

Flow cytometric analysis of the LDLR expressed on the cell surface was conducted as previously described[53] with slight modification. Following transfection or transduction for 48 h, the medium was changed to serum-free Opti-MEM and incubated at 37°C for 24 h, and then HepG2, Hepa1–6, and AML12 cells were digested with trypsin, detached by scraping, washed with PBS, and collected in a 1.5 mL tube, then fixed in 200 mL 4% (w/v) paraformaldehyde in PBS for 5 min at room temperature. Cells were incubated with 200 mL of 0.1% Tween in PBS (PBS-T) and blocked with 10% goat serum in 0.3 M glycine in PBS for 30 min, then incubated with rabbit anti-LDLR monoclonal antibody (catalog #ab52818; 1:100) for 30 min at room temperature, followed by incubation with Alexa Fluor 488-conjugated goat anti-rabbit IgG (catalog #D110061; 1:200) or Alexa Fluor 555-conjugated goat anti-rabbit IgG (catalog #D110070; 1:200) for 30 min at room temperature. After washing, detection for Alexa Fluor 488 was performed directly on a Guava Easy Cyte flow cytometer (Merck Millipore) at an excitation wavelength of 488 nm and an emission wavelength of 525 nm, while Alexa Fluor 555 was detected at an excitation wavelength of 555 nm and an emission wavelength of 580 nm. The levels of LDLR on the cell surface were analyzed using FlowJo version 7.6 with 10,000 cells.

### Immunofluorescence assay

Detection of IDOL and LDLR in HepG2, Hepa1–6, and AML12 cells and liver tissues by immunofluorescence was performed as previously described[52,53] with minor modification. Briefly, after treatment, HepG2 and Hepa1–6 cells were rinsed 3 times with PBS for 5 min and fixed in 4% (w/v) paraformaldehyde in PBS for 30 min. Liver tissues were fixed in 4% (w/v) paraformaldehyde in PBS at 4°C for 48 h, embedded in paraffin, and sliced at 4 mm thickness. After deparaffinization and hydration, tissue sections were pretreated by heating for 20 min in sodium citrate solution (0.01 M, pH 6.0) in a 95°C water bath for antigen retrieval. Thereafter, the cells or tissue sections were blocked with 10% (v/v) goat serum in PBS-T for 1 h and incubated with rabbit anti-IDOL antibody (1:100; catalog #D126668) or rabbit anti-LDLR antibody (1:100; catalog #ab52818) overnight at 4°C, followed by incubation with Alexa Fluor 488-conjugated goat anti-rabbit IgG (1:200; catalog #D110061) or Alexa Fluor 555-conjugated goat anti-rabbit IgG (1:200; catalog #D110070) for 1 h at room temperature and counter-stained with DAPI (Key GEN Bio TECH, Nanjing, China) to show cell nucleus. Images were acquired using a Zeiss LSM700 confocal microscope (Zeiss, Oberkochen, Germany).

### Homology modeling and molecular docking

The 3D structure of CS8 peptide-incorporated VSV-G (GenBank: OQ561788) was built on the basis of the wild-type VSV-G crystal structure (PDB: 5I2S) using I-TASSER (http://zhanglab.ccmb.med.umich.edu/I-TASSER/).[54–56] The modeled structure of CS8 peptide-incorporated VSV-G was docked with the crystal structure of LRP1 CR.7 (PDB: 1J8E) using HawkDock web server (http://cadd.zju.edu.cn/hawkdock/),[57,58] and the binding free energy ($\Delta G_{bind}$) of the ten top-ranked binding poses was calculated in HawkDock web server using the MM/GBSA method. Then, the docked poses with the lowest binding free energies were selected as the best conformations, and the key interacting residues in those docked complexes were analyzed and mapped using Molecular Operating Environment for Windows.

### Mice

All animal experiments were approved by the ethics committee of China Pharmaceutical University (#201601179, October 19, 2016) and conformed to the Guide for the Care and Use of Laboratory Animals published by the National Institutes of Health. Six-week-old male C57BL/6 mice were obtained from Qinglongshan Experimental Animal Breeding Farm (SCXK [Su] 2020-0001, Nanjing, China) and maintained on a 12 h light/dark cycle at 25°C.

### *In vivo* distribution of liver-targeted lentiviral IDOL-shRNA

To enable assessment of biodistribution, liver-targeted CS8-LV-shIDOL was injected into the tail veins of C57/BL6 mice at a dose of $1 \times 10^8$ TU per mouse. Control mice were injected with an equivalent dose of wild-type lentivirus LV-shIDOL or saline. One week after injection, mice were sacrificed, and the fluorescence intensity in five different organs (heart, liver, kidney, lung, and spleen) was detected using IVIS Kinetic Bioluminescence imager (PerkinElmer). Subsequently, tissues were fixed in 4% (w/v) paraformaldehyde and embedded in paraffin for histological examination. Tissue sections were prepared, and ZsGreen expression was analyzed immediately using a fluorescence microscope (AX10).

### *In vivo* toxicity evaluation

C57BL/6 mice were intravenously administered with the equivalent dose ($1 \times 10^8$ TU per mouse) of either LV-shIDOL or CS8-LV-shIDOL. Control mice were injected with saline. One week after injection, mice were euthanized, and blood and major organs were harvested. Assay kits (Beyotime) were used to measure serum levels of ALT, AST, ALP, plasma urea, and CREA. Organs were fixed in

paraformaldehyde, sectioned, and stained with H&E for morphological analysis.

#### *In vivo* IDOL-shRNA treatment

After acclimation for one week, mice were randomly divided into five groups (n = 8 each): (1) normal group: normal mice fed on a low fat diet (catalog #TP28602; TrophicDiet, Nantong, China) treated with saline; (2) model group: P407-induced hyperlipidemic mice fed an atherogenic diet (catalog #TP28600; TrophicDiet) treated with saline; (3) negative control shRNA group: P407-induced hyperlipidemic mice fed an atherogenic diet treated with LV-shNC; (4) LV-shIDOL group: P407-induced hyperlipidemic mice fed an atherogenic diet treated with LV-shIDOL; and (5) CS8-LV-shIDOL group: P407-induced hyperlipidemic mice fed an atherogenic diet treated with CS8-LV-shIDOL. In order to induce atherosclerotic lesions, all animals except the normal group were fed an atherogenic diet (containing 20% fat, 1.5% cholesterol, and 0.5% cholic acid; catalog #TP28600; TrophicDiet) were simultaneously treated every third day with an intraperitoneal injection of P407 (0.5 g $kg^{-1}$) for a time period of 16 weeks. For negative control shRNA, LV-shIDOL, and CS8-LV-shIDOL groups, mice were received corresponding LVs ($1 \times 10^8$ TU per mouse) via tail-vein injection every eight weeks, while mice in the normal control group and model group were injected with saline (vehicle). Twenty-four hours after the final injection of P407, all mice were fasted for 8 h and then euthanized for collection of blood sample, aorta, lung, heart, liver, spleen, and kidney. Liver tissues were dissected and further analyzed using western blot. Heart, liver, spleen, lung, and kidney were fixed in 4% (w/v) paraformaldehyde and embedded in paraffin for histological examination. In addition, liver tissues were also embedded in optimal cutting temperature (OCT) freezing medium and stained with H&E for morphological analysis and oil red O to detect lipid accumulation. To determine lesions in aortic root, frozen sections of aortic root were prepared and then stained with oil red O solution.

#### Statistical analysis

All values are presented as mean ± SEM and were analyzed using Prism 6.0 software (GraphPad Software, La Jolla, CA). Normality of data was determined using the Kolmogorov-Smirnov test. Normally distributed variables between 2 independent groups were compared using unpaired Student's t tests, whereas non-normally distributed variables were analyzed using the Mann-Whitney U test. A two-tailed p value of $<0.05$ was considered to indicate statistical significance.

## DATA AND CODE AVAILABILITY

All data associated with this study are present in the paper or the supplemental information.

## SUPPLEMENTAL INFORMATION

Supplemental information can be found online at https://doi.org/10.1016/j.omtn.2024.102115.

## ACKNOWLEDGMENTS

This work was supported by National Fund for Major Projects of China (2009ZX09103-653, 2013ZX09301303-006, and 2018ZX09301035), the Priority Academic Program Development of Jiangsu Higher Education Institutions (PAPD), the National Fund for Fostering Talents of Basic Science (NFFTBS; 3050040016), and the China Pharmaceutical University "Double First-Class" project (CPU2018GY15).

## AUTHOR CONTRIBUTIONS

W.W. and X.C. designed and performed research, analyzed data, and wrote the manuscript. J.C., M.X., and L.Y. performed research. W.Z. and S.Y. designed experiments. S.T. designed research, wrote the manuscript, and coordinated the project.

## DECLARATION OF INTERESTS

The authors declare no competing interests.

## REFERENCES


1. Moreira, A.S., Cavaco, D.G., Faria, T.Q., Alves, P.M., Carrondo, M.J.T., and Peixoto, C. (2021). Advances in Lentivirus Purification. Biotechnol. J. *16*, e2000019.
2. Milone, M.C., and O'Doherty, U. (2018). Clinical use of lentiviral vectors. Leukemia *32*, 1529–1541.
3. Martínez-Molina, E., Chocarro-Wrona, C., Martínez-Moreno, D., Marchal, J.A., and Boulaiz, H. (2020). Large-scale production of lentiviral vectors: current perspectives and challenges. Pharmaceutics *12*, 1051.
4. Palfi, S., Gurruchaga, J.M., Ralph, G.S., Lepetit, H., Lavisse, S., Buttery, P.C., Watts, C., Miskin, J., Kelleher, M., Deeley, S., et al. (2014). Long-term safety and tolerability of ProSavin, a lentiviral vector-based gene therapy for Parkinson's disease: a dose escalation, open-label, phase 1/2 trial. Lancet *383*, 1138–1146.
5. Campochiaro, P.A., Lauer, A.K., Sohn, E.H., Mir, T.A., Naylor, S., Anderton, M.C., Kelleher, M., Harrop, R., Ellis, S., and Mitrophanous, K.A. (2017). Lentiviral vector gene transfer of endostatin/angiostatin for macular degeneration (GEM) study. Hum. Gene Ther. *28*, 99–111.
6. Hutson, T.H., Foster, E., Moon, L.D.F., and Yáñez-Muñoz, R.J. (2014). Lentiviral vector-mediated RNA silencing in the central nervous system. Hum. Gene Ther. Methods *25*, 14–32.
7. Alfranca, A., Campanero, M.R., and Redondo, J.M. (2018). New methods for disease modeling using lentiviral vectors. Trends Mol. Med. *24*, 825–837.
8. Gutierrez-Guerrero, A., Cosset, F.L., and Verhoeyen, E. (2020). Lentiviral Vector Pseudotypes: Precious Tools to Improve Gene Modification of Hematopoietic Cells for Research and Gene Therapy. Viruses *12*, 1016.
9. Roche, S., Bressanelli, S., Rey, F.A., and Gaudin, Y. (2006). Crystal structure of the low-pH form of the vesicular stomatitis virus glycoprotein G. Science *313*, 187–191.
10. Roche, S., Rey, F.A., Gaudin, Y., and Bressanelli, S. (2007). Structure of the prefusion form of the vesicular stomatitis virus glycoprotein. G. Science *315*, 843–848.
11. Kappe, S.H.I., Buscaglia, C.A., and Nussenzweig, V. (2004). Plasmodium sporozoite molecular cell biology. Annu. Rev. Cell Dev. Biol. *20*, 29–59.
12. Garcia, J.E., Puentes, A., and Patarroyo, M.E. (2006). Developmental biology of sporozoite-host interactions in Plasmodium falciparum malaria: implications for vaccine design. Clin. Microbiol. Rev. *19*, 686–707.
13. Suarez, J.E., Urquiza, M., Puentes, A., Garcia, J.E., Curtidor, H., Ocampo, M., Lopez, R., Rodriguez, L.E., Vera, R., Cubillos, M., et al. (2001). Plasmodium falciparum circumsporozoite (CS) protein peptides specifically bind to HepG2 cells. Vaccine *19*, 4487–4495.
14. Olsson, U., Camejo, G., Hurt-Camejo, E., Elfsber, K., Wiklund, O., and Bondjers, G. (1997). Possible Functional Interactions of Apolipoprotein B-100 Segments That Associate With Cell Proteoglycans and the Apo B/E Receptor. Arterioscler. Thromb. Vasc. Biol. *17*, 149–155.

15. Zelcer, N., Hong, C., Boyadjian, R., and Tontonoz, P. (2009). LXR regulates cholesterol uptake through Idol-dependent ubiquitination of the LDL receptor. Science *325*, 100–104.

16. Calkin, A.C., Goult, B.T., Zhang, L., Fairall, L., Hong, C., Schwabe, J.W.R., and Tontonoz, P. (2011). FERM-dependent E3 ligase recognition is a conserved mechanism for targeted degradation of lipoprotein receptors. Proc. Natl. Acad. Sci. USA *108*, 20107–20112.

17. Zhang, L., Fairall, L., Goult, B.T., Calkin, A.C., Hong, C., Millard, C.J., Tontonoz, P., and Schwabe, J.W.R. (2011). The IDOL-UBE2D complex mediates sterol-dependent degradation of the LDL receptor. Genes Dev. *25*, 1262–1274.

18. Scotti, E., Hong, C., Yoshinaga, Y., Tu, Y., Hu, Y., Zelcer, N., Boyadjian, R., de Jong, P.J., Young, S.G., Fong, L.G., and Tontonoz, P. (2011). Targeted disruption of the idol gene alters cellular regulation of the low-density lipoprotein receptor by sterols and liver x receptor agonists. Mol. Cell Biol. *31*, 1885–1893.

19. Sorrentino, V., Nelson, J.K., Maspero, E., Marques, A.R.A., Scheer, L., Polo, S., and Zelcer, N. (2013). The LXR-IDOL axis defines a clathrin-caveolae-and dynamin-independent endocytic route for LDLR internalization and lysosomal degradation. J. Lipid Res. *54*, 2174–2184.

20. Scotti, E., Calamai, M., Goulbourne, C.N., Zhang, L., Hong, C., Lin, R.R., Choi, J., Pilch, P.F., Fong, L.G., Zou, P., et al. (2013). IDOL stimulates clathrin-independent endocytosis and multivesicular body-mediated lysosomal degradation of the low-density lipoprotein receptor. Mol. Cell Biol. *33*, 1503–1514.

21. Zhang, C.P., Tian, Y., Zhang, M., Tuo, Q.H., Chen, J.X., and Liao, D.F. (2016). IDOL, inducible degrader of low-density lipoprotein receptor, serves as a potential therapeutic target for dyslipidemia. Med. Hypotheses *86*, 138–142.

22. van Loon, N.M., Lindholm, D., and Zelcer, N. (2019). The E3 ubiquitin ligase inducible degrader of the LDL receptor/myosin light chain interacting protein in health and disease. Curr. Opin. Lipidol. *30*, 192–197.

23. Leitch, E.K., Elumalai, N., Fridén-Saxin, M., Dahl, G., Wan, P., Clarkson, P., Valeur, E., Pairaudeau, G., Boyd, H., and Tavassoli, A. (2018). Inhibition of low-density lipoprotein receptor degradation with a cyclic peptide that disrupts the homodimerization of IDOL E3 ubiquitin ligase. Chem. Sci. *9*, 5957–5966.

24. Meister, G., and Tuschl, T. (2004). Mechanisms of gene silencing by double-stranded RNA. Nature *431*, 343–349.

25. Vaishnaw, A.K., Gollob, J., Gamba-Vitalo, C., Hutabarat, R., Sah, D., Meyers, R., de Fougerolles, T., and Maraganore, J. (2010). A status report on RNAi therapeutics. Silence *1*, 14.

26. Ray, K.K., Landmesser, U., Leiter, L.A., Kallend, D., Dufour, R., Karakas, M., Hall, T., Troquay, R.P.T., Turner, T., Visseren, F.L.J., et al. (2017). Inclisiran in patients at high cardiovascular risk with elevated LDL cholesterol. N. Engl. J. Med. *376*, 1430–1440.

27. Ozcan, G., Ozpolat, B., Coleman, R.L., Sood, A.K., and Lopez-Berestein, G. (2015). Preclinical and clinical development of siRNA-based therapeutics. Adv. Drug Deliv. Rev. *87*, 108–119.

28. Ammayappan, A., Peng, K.W., and Russell, S.J. (2013). Characteristics of oncolytic vesicular stomatitis virus displaying tumor-targeting ligands. J. Virol. *87*, 13543–13555.

29. Shi, W., Arnold, G.S., and Bartlett, J.S. (2001). Insertional mutagenesis of the adeno-associated virus type 2 (AAV2) capsid gene and generation of AAV2 vectors targeted to alternative cell-surface receptors. Hum. Gene Ther. *12*, 1697–1711.

30. Moore, C.B., Guthrie, E.H., Huang, M.T.H., and Taxman, D.J. (2010). Short hairpin RNA (shRNA): design, delivery, and assessment of gene knockdown. Methods Mol. Biol. *629*, 141–158.

31. Paigen, B., Morrow, A., Holmes, P.A., Mitchell, D., and Williams, R.A. (1987). Quantitative assessment of atherosclerotic lesions in mice. Atherosclerosis *68*, 231–240.

32. Palmer, W.K., Emeson, E.E., and Johnston, T.P. (1998). Poloxamer 407-induced atherogenesis in the C57BL/6 mouse. Atherosclerosis *136*, 115–123.

33. Zhou, X., Johnston, T.P., Johansson, D., Parini, P., Funa, K., Svensson, J., and Hansson, G.K. (2009). Hypercholesterolemia leads to elevated TGF-beta1 activity and T helper 3-dependent autoimmune responses in atherosclerotic mice. Atherosclerosis *204*, 381–387.

34. Finkelshtein, D., Werman, A., Novick, D., Barak, S., and Rubinstein, M. (2013). LDL receptor and its family members serve as the cellular receptors for vesicular stomatitis virus. Proc. Natl. Acad. Sci. USA *110*, 7306–7311.

35. High, K.A., and Roncarolo, M.G. (2019). Gene therapy. N. Engl. J. Med. *381*, 455–464.

36. Singer, O., and Verma, I.M. (2008). Applications of lentiviral vectors for shRNA delivery and transgenesis. Curr. Gene Ther. *8*, 483–488.

37. Anguela, X.M., and High, K.A. (2019). Entering the modern era of gene therapy. Annu. Rev. Med. *70*, 273–288.

38. Pan, D., Gunther, R., Duan, W., Wendell, S., Kaemmerer, W., Kafri, T., Verma, I.M., and Whitley, C.B. (2002). Biodistribution and toxicity studies of VSVG-pseudotyped lentiviral vector after intravenous administration in mice with the observation of in vivo transduction of bone marrow. Mol. Ther. *6*, 19–29.

39. Fong, L.G., Bonney, E., Kosek, J.C., and Cooper, A.D. (1989). Immunohistochemical localization of low density lipoprotein receptors in adrenal gland, liver, and intestine. J. Clin. Invest. *84*, 847–856.

40. Herz, J., and Bock, H.H. (2002). Lipoprotein receptors in the nervous system. Annu. Rev. Biochem. *71*, 405–434.

41. Shakibaei, M., and Frevert, U. (1996). Dual interaction of the malaria circumsporozoite protein with the low density lipoprotein receptor-related Protein (LRP) and heparan sulfate proteoglycans. J. Exp. Med. *184*, 1699–1711.

42. van de Sluis, B., Wijers, M., and Herz, J. (2017). News on the molecular regulation and function of hepatic low-density lipoprotein receptor and LDLR-related protein 1. Curr. Opin. Lipidol. *28*, 241–247.

43. Herz, J., Kowal, R.C., Ho, Y.K., Brown, M.S., and Goldstein, J.L. (1990). Low density lipoprotein receptor-related protein mediates endocytosis of monoclonal antibodies in cultured cells and rabbit liver. J. Biol. Chem. *265*, 21355–21362.

44. Ludtke, J.J., Sokoloff, A.V., Wong, S., Zhang, G., Strickland, D.K., and Wolff, J.A. (2009). Peptide-mediated targeting of hepatocytes via low density lipoprotein receptor-related protein (LRP). Drug Deliv. *16*, 268–273.

45. Vert, J.P., Foveau, N., Lajaunie, C., and Vandenbrouck, Y. (2006). An accurate and interpretable model for siRNA efficacy prediction. BMC Bioinf. *7*, 520.

46. Reuter, J.S., and Mathews, D.H. (2010). RNAstructure: software for RNA secondary structure prediction and analysis. BMC Bioinf. *11*, 129.

47. Kutner, R.H., Zhang, X.Y., and Reiser, J. (2009). Production, concentration and titration of pseudotyped HIV-1-based lentiviral vectors. Nat. Protoc. *4*, 495–505.

48. Rubinson, D.A., Dillon, C.P., Kwiatkowski, A.V., Sievers, C., Yang, L., Kopinja, J., Rooney, D.L., Zhang, M., Ihrig, M.M., McManus, M.T., et al. (2003). A lentivirus-based system to functionally silence genes in primary mammalian cells, stem cells and transgenic mice by RNA interference. Nat. Genet. *33*, 401–406.

49. Nasri, M., Karimi, A., and Allahbakhshian Farsani, M. (2014). Production, purification and titration of a lentivirus-based vector for gene delivery purposes. Cytotechnology *66*, 1031–1038.

50. Gu, L., Ye, P., Li, H., Wang, Y., Xu, Y., Tian, Q., Lei, G., Zhao, C., Gao, Z., Zhao, W., and Tan, S. (2019). Lunasin attenuates oxidant-induced endothelial injury and inhibits atherosclerotic plaque progression in ApoE(-/-) mice by up-regulating heme oxygenase-1 via PI3K/Akt/Nrf2/ARE pathway. Faseb. J. *33*, 4836–4850.

51. Livak, K.J., and Schmittgen, T.D. (2001). Analysis of Relative Gene Expression Data Using Real-Time Quantitative PCR and the 2−ΔΔCT Method. Methods *25*, 402–408.

52. Ly, K., Saavedra, Y.G.L., Canuel, M., Routhier, S., Desjardins, R., Hamelin, J., Mayne, J., Lazure, C., Seidah, N.G., and Day, R. (2014). Annexin A2 reduces PCSK9 protein levels via a translational mechanism and interacts with the M1 and M2 domains of PCSK9. J. Biol. Chem. *289*, 17732–17746.

53. Xu, Y., Gao, J., Gong, Y., Chen, M., Chen, J., Zhao, W., and Tan, S. (2020). Hsa-miR-140-5p down-regulates LDL receptor and attenuates LDL-C uptake in human hepatocytes. Atherosclerosis *297*, 111–119.

54. Wu, S., Skolnick, J., and Zhang, Y. (2007). Ab initio modeling of small proteins by iterative TASSER simulations. BMC Biol. *5*, 17.

55. Zhang, Y. (2008). I-TASSER server for protein 3D structure prediction. BMC Bioinf. *9*, 40.

56. Zhang, Y. (2007). Template-based modeling and free modeling by I-TASSER in CASP7. Proteins *69* (*Suppl 8*), 108–117.

57. Yan, Y., Tao, H., He, J., and Huang, S.Y. (2020). The HDOCK server for integrated protein-protein docking. Nat. Protoc. *15*, 1829–1852.

58. Weng, G., Wang, E., Wang, Z., Liu, H., Zhu, F., Li, D., and Hou, T. (2019). HawkDock: a web server to predict and analyze the protein-protein complex based on computational docking and MM/GBSA. Nucleic Acids Res. *47*, W322–W330.

**OMTN, Volume *35***

## Supplemental information

# Engineering lentivirus envelope VSV-G for liver targeted delivery of IDOL-shRNA to ameliorate hypercholesterolemia and atherosclerosis

**Wei Wang, Xuemei Chen, Jiali Chen, Menglong Xu, Ying Liu, Shijie Yang, Wenfeng Zhao, and Shuhua Tan**

# Supplemental Data

## Supplemental Figures

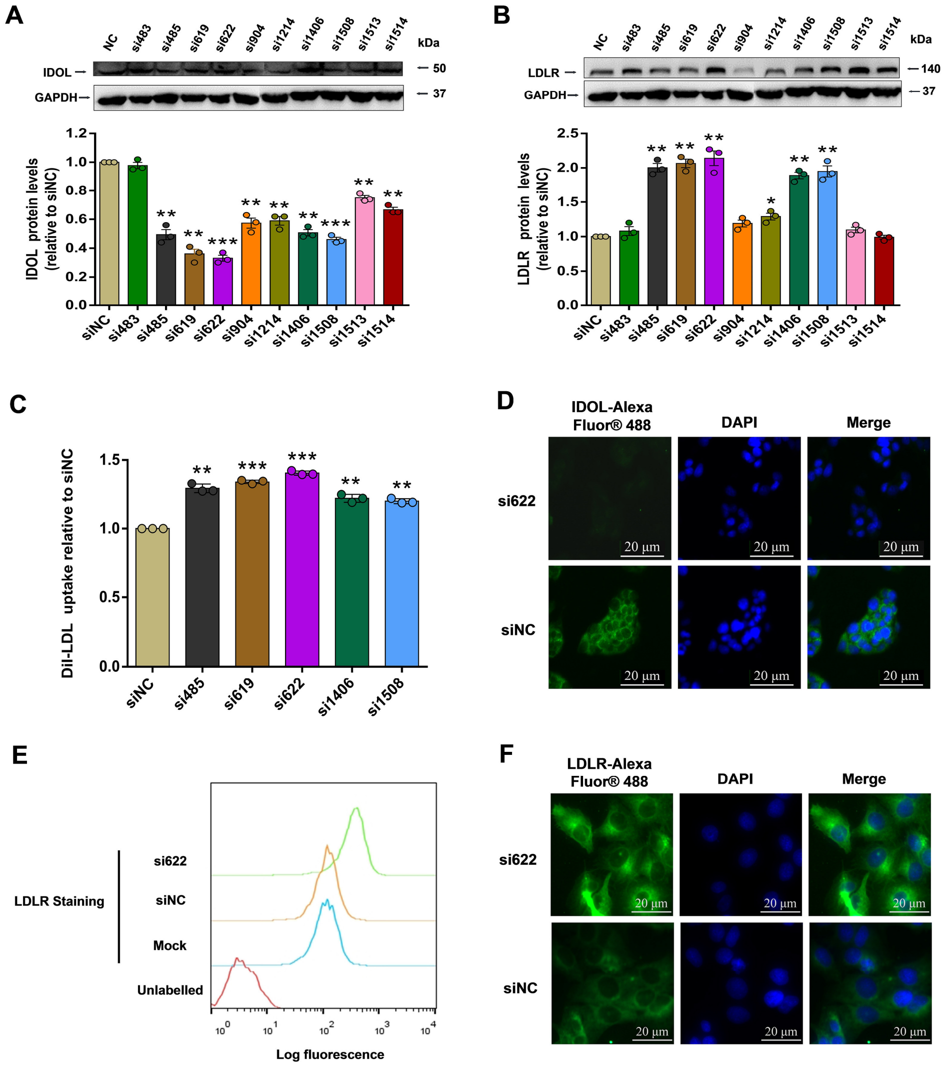


**Fig. S1. Screening of IDOL siRNAs in human HepG2 hepatocytes.** (**A** to **C**) Identification of efficacious IDOL siRNA in vitro. HepG2 cells were transfected with 30 nM siNC (negative control) and candidate siRNAs. 72h later, the levels of LDLR protein were analyzed by western blot normalized to that of the siNC group (**A** and **B**). After transfection with 30 nM siIDOL-485, siIDOL-619, siIDOL-622, siIDOL-1406 and siIDOL-1508 for 72 h, DiI-LDL uptake levels were measured using a Multimode Reader normalized to that of the siNC group (**C**). (**D** to **F**) Effects of siIDOL-622 on IDOL and LDLR expression in HepG2 cells. After transfection with 30 nM siNC and siIDOL-622 for 72h, IDOL protein levels were visualized by Immunofluorescence (**D**). The cell surface LDLR protein levels were determined by flow cytometry (**E**) and evaluated by Immunofluorescence (**F**). * $p<0.05$, ** $p<0.01$, *** $p<0.001$ vs siNC treatment group (unpaired Student's t-test). Results are given as the means±SEM of three independent experiments.

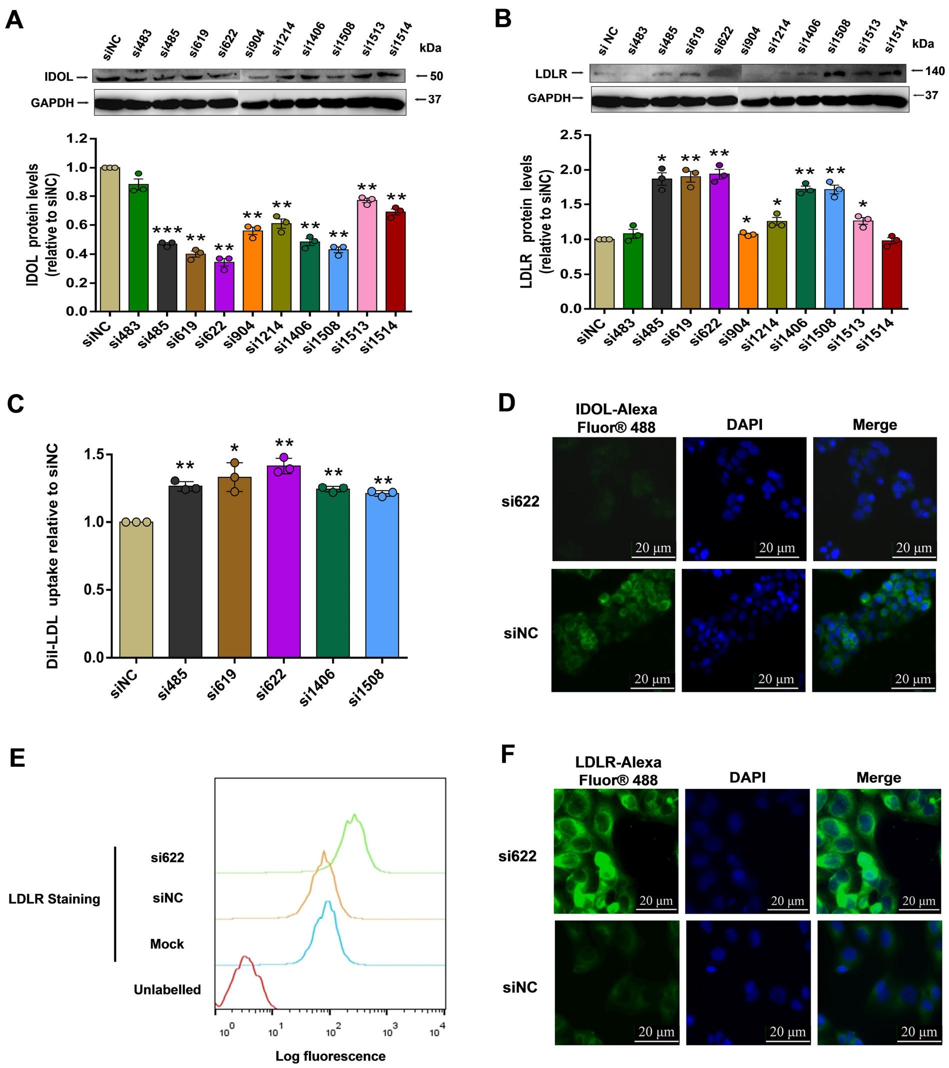

**Fig. S2. Efficient siRNA-mediated knockdown of IDOL increases LDLR levels in mouse hepatic cells.** (**A** and **B**) Hepa 1-6 cells were transfected with 30 nM siNC (negative control) and various IDOL siRNAs. 72h later, the levels of LDLR protein were analyzed by western blot normalized to that of the siNC group. (**C** to **F**) Effects of IDOL siRNAs on LDLR expression and DiI-LDL uptake in mouse hepatocytes. After transfection with 30 nM siIDOL-485, siIDOL-619, siIDOL-622, siIDOL-1406 and siIDOL-1508 for 72 h, DiI-LDL uptake levels were measured using a Multimode Reader normalized to that of the siNC group (**C**). Hepa 1-6 cells were transfected with 30 nM siNC and siIDOL-622. 72h later, protein levels of IDOL were evaluated by immunofluorescence (**D**). The cell surface LDLR protein levels were determined by flow cytometry (**E**) and visualized by Immunofluorescence (**F**). * $p<0.05$, ** $p<0.01$, *** $p<0.001$ vs siNC treatment group (unpaired Student's t-test). Results are given as the means±SEM of three independent experiments.

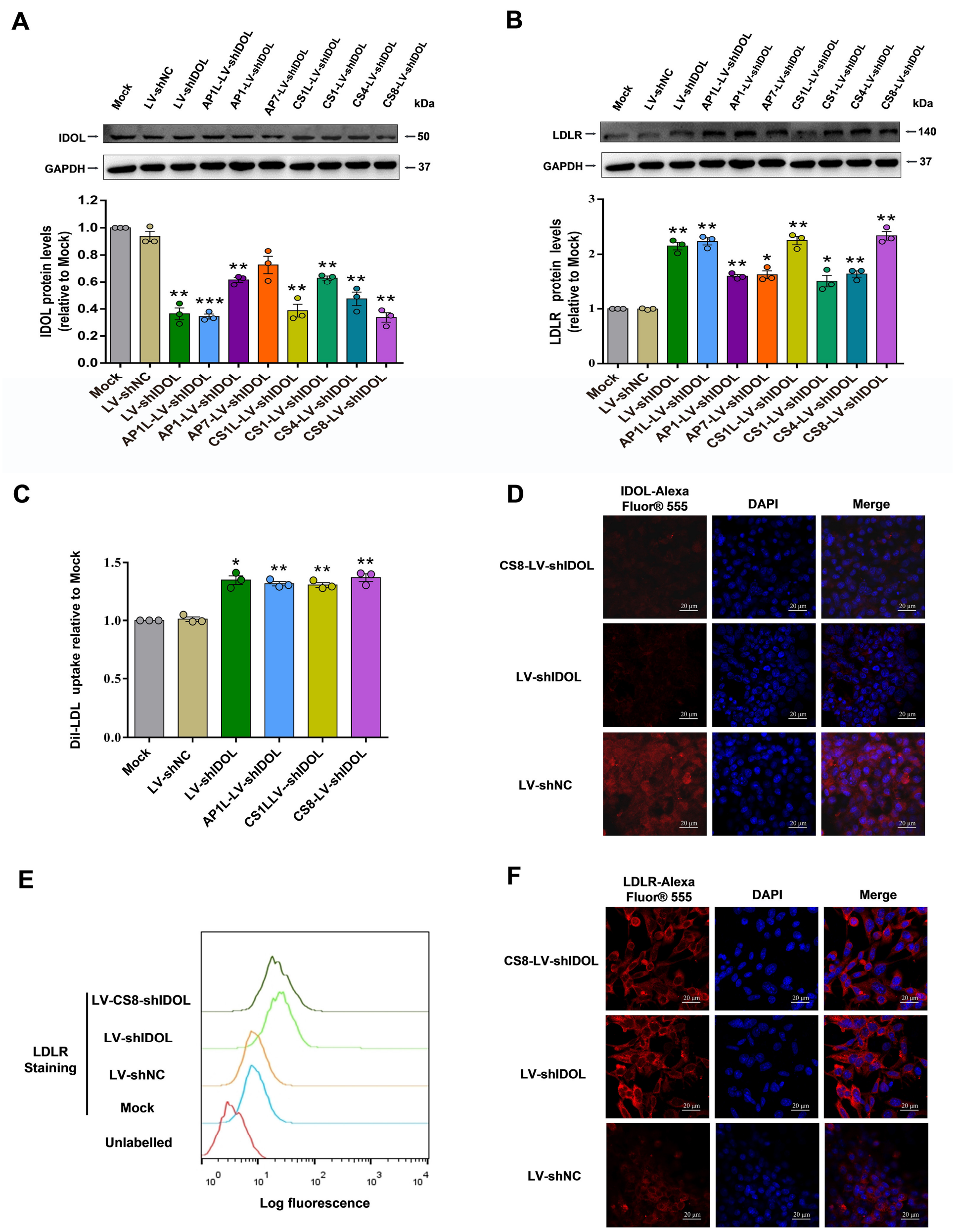


**Fig. S3. Efficient shRNA-mediated silencing of IDOL elevates LDLR levels in mouse hepatocytes.** (**A** and **B**) Hepa 1-6 cells were transducted with LV-shNC (negative control), LV-shIDOL (wild type control) and lentiviral IDOL shRNAs with different VSVG variants. 72h later, the levels of LDLR protein were analyzed by western blot normalized to that of the Mock group. (**C** to **F**) Effects of IDOL shRNAs on LDLR expression and DiI-LDL uptake in mouse hepatocytes. After transduction with LV-shIDOL, AP1L-LV-shIDOL, CS1L-LV-shIDOL and CS8-LV-shIDOL for 72 h, DiI-LDL uptake levels were measured using a Multimode Reader normalized to that of the Mock group (**C**). Hepa 1-6 cells were transducted with LV-shNC, LV-shIDOL and CS8-LV-shIDOL. 72h later, protein levels of IDOL were evaluated by immunofluorescence (**D**). The cell surface LDLR protein levels were determined by flow cytometry (**E**) and visualized by Immunofluorescence (**F**). * $p<0.05$, ** $p<0.01$, *** $p<0.001$ vs mock group (unpaired Student's t-test). Results are given as the means±SEM of three independent experiments.

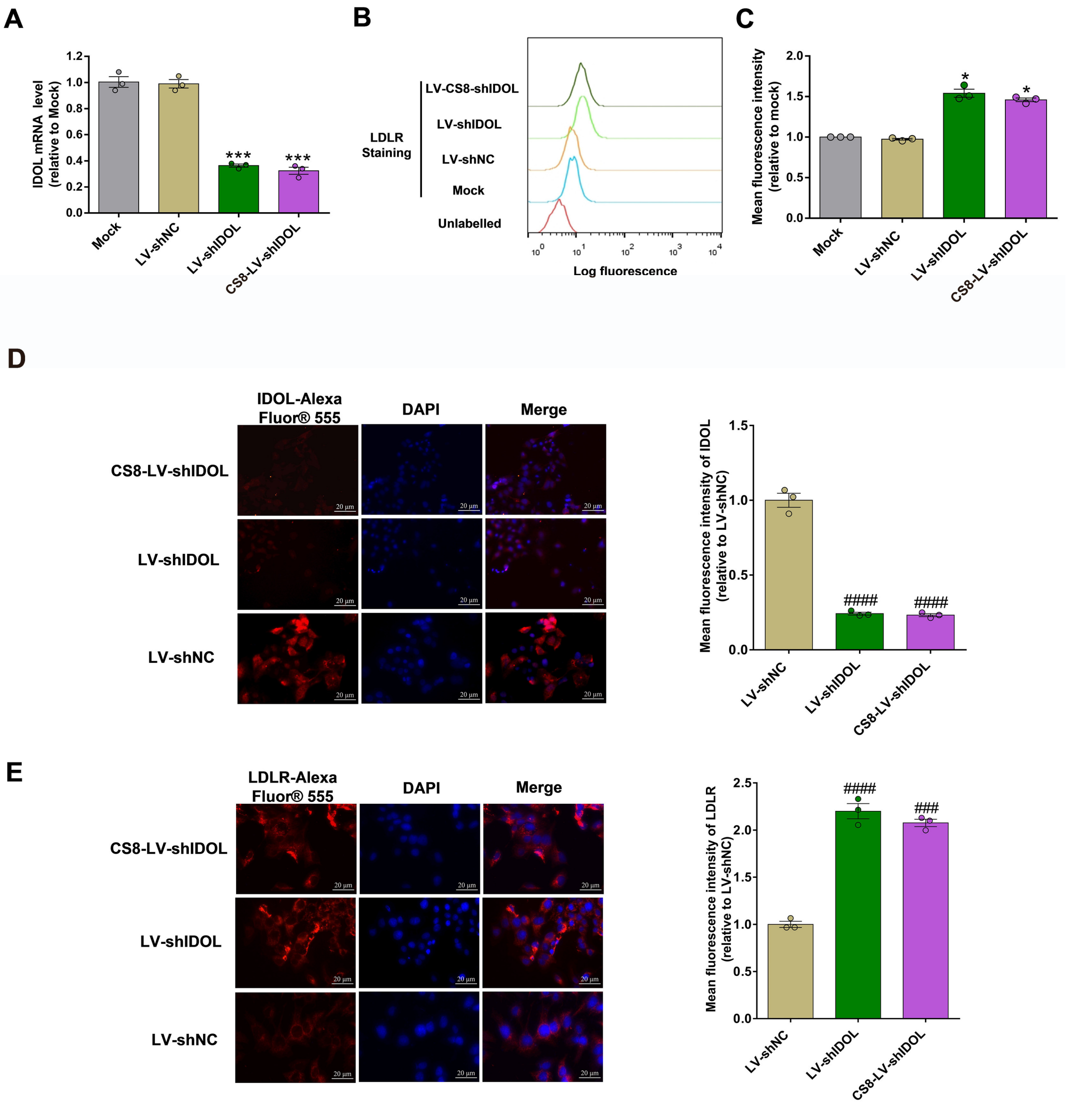


**Fig. S4. Efficient shRNA-mediated silencing of IDOL elevates LDLR levels in LO2 cells.** (**A**) LO2 cells were transducted with LV-shNC (negative control), LV-shIDOL (wild type control) and CS8-LV-shIDOL. After 48 h, the levels of IDOL mRNA in LO2 cells were quantifed by qRT-PCR. (**B-E**) Effects of IDOL shRNAs on LDLR expression in LO2 cells. LO2 cells were transducted with LV-shNC, LV-shIDOL and CS8-LV-shIDOL. 72h later, protein levels of IDOL were evaluated by immunofluorescence (**D**). The cell surface LDLR protein levels were determined by flow cytometry (**B,C**) and visualized by Immunofluorescence (**E**). * $p<0.05$, *** $p<0.001$ vs mock group; ### $P< 0.001$, #### $P< 0.0001$ vs. LV-shNC group (unpaired Student's t-test). Results are given as the means±SEM of three independent experiments.

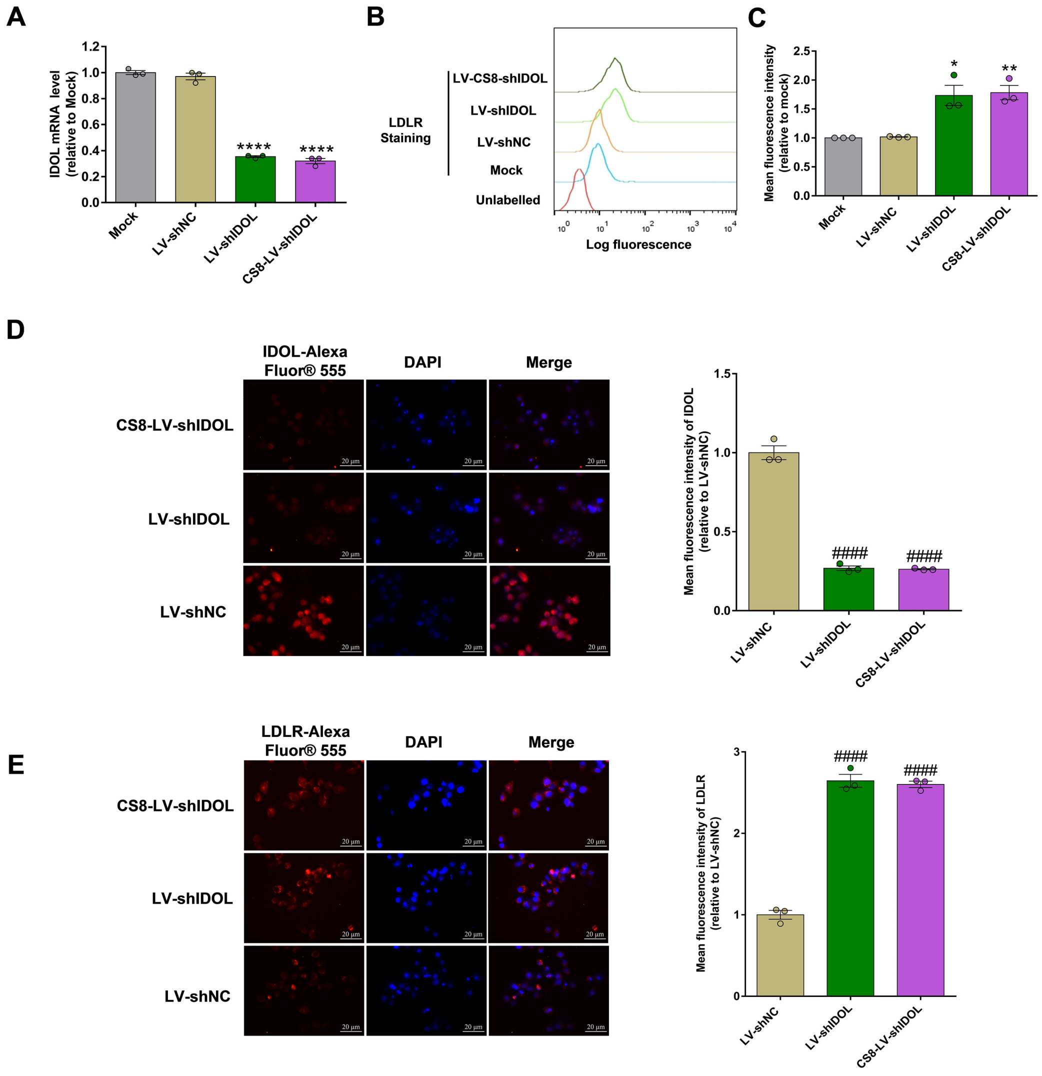


**Fig. S5. Efficient shRNA-mediated silencing of IDOL elevates LDLR levels in AML12 cells.** (**A**) AML12 cells were transducted with LV-shNC (negative control), LV-shIDOL (wild type control) and CS8-LV-shIDOL. After 48 h, the levels of IDOL mRNA in AML12 cells were quantifed by qRT-PCR. (**B-E**) Effects of IDOL shRNAs on LDLR expression in AML12 cells. AML12 cells were transducted with LV-shNC, LV-shIDOL and CS8-LV-shIDOL. 72h later, protein levels of IDOL were evaluated by immunofluorescence (**D**). The cell surface LDLR protein levels were determined by flow cytometry (**B,C**) and visualized by Immunofluorescence (**E**). * p<0.05, ** P<0.01, and **** p<0.0001 vs mock group; #### P< 0.0001 vs. LV-shNC group (unpaired Student's t-test). Results are given as the means±SEM of three independent experiments.

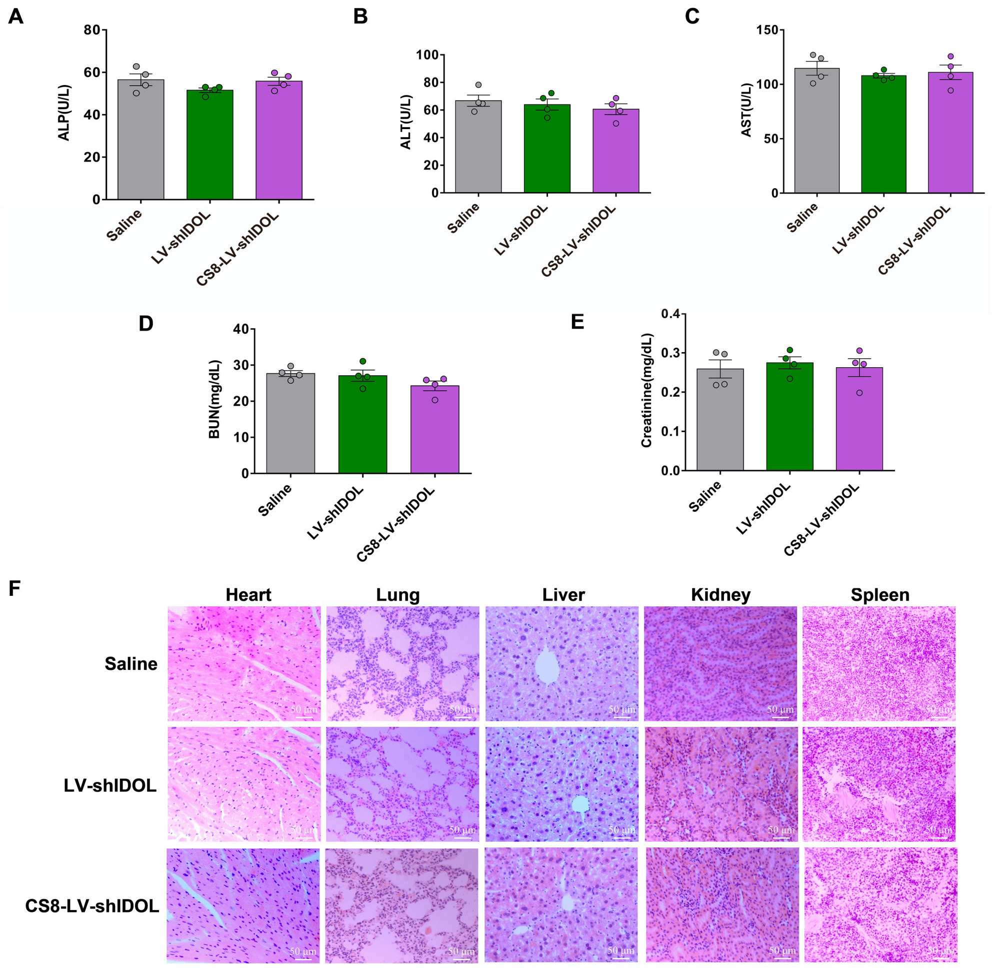


**Fig. S6. In vivo biosafety analysis of CS8-LV-shIDOL**. (**A-E**) Serum alanine aminotransferase (ALT), aspartate aminotransferase (AST), alkaline phosphatase (ALP), plasma urea (BUN) and creatinine were measured. Data represent mean values ± SEM. (F) Hematoxylin and eosin stainin images obtained from the major organs of LV-shIDOL, CS8-LV-shIDOL and saline treated mice. Scale bars, 50 µm.

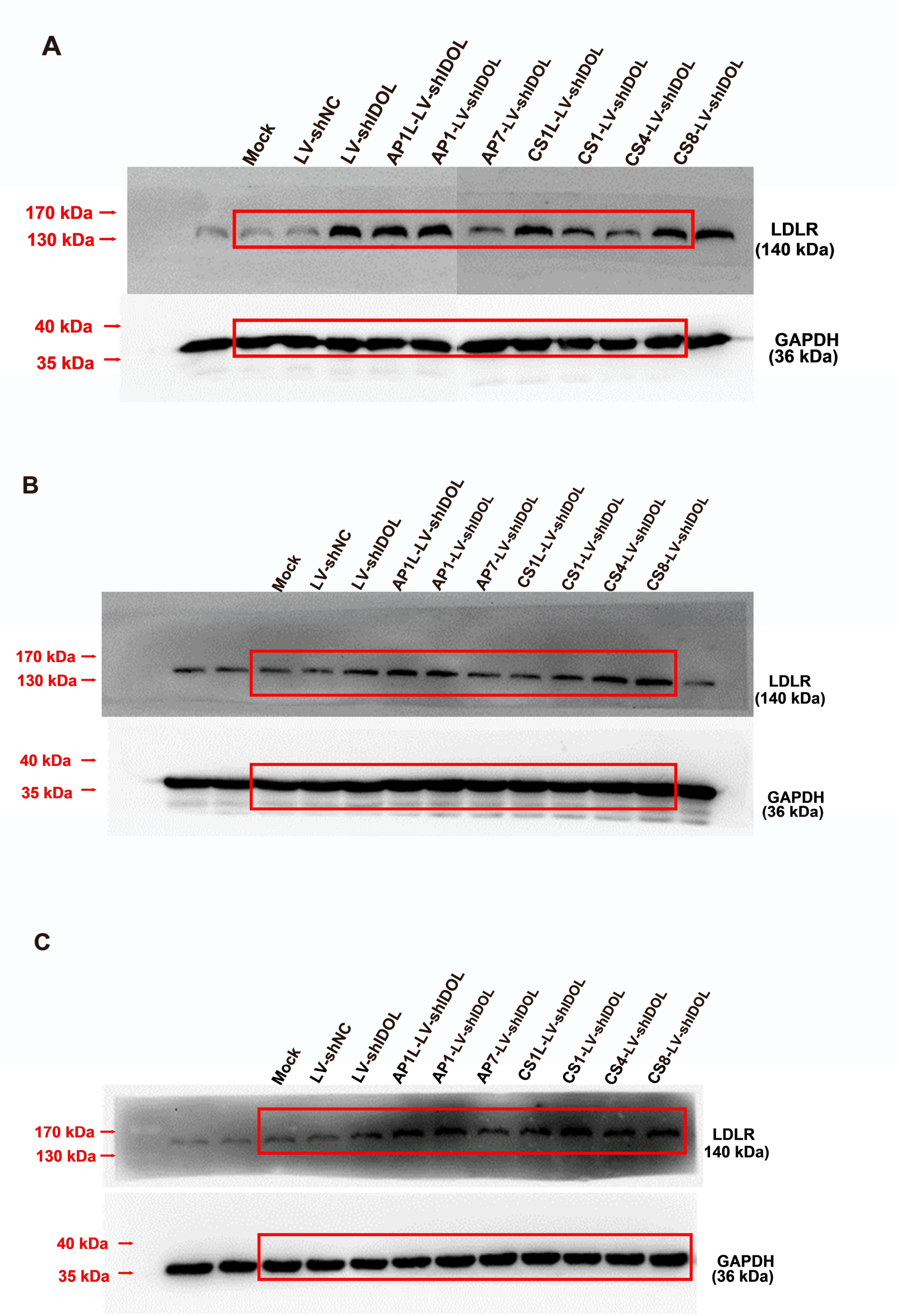


**Fig. S7. Full scans of blots presented in cropped format for Figure 4C.** Red boxes show the lanes included in the main text.

**Supplemental Tables:**

**Table S1. Summary of various ligand insertion sites on VSV-G.** The amino acid numbering used in this study was taken from that for the VSV-G crystal structure. Insertions were made into the wild type lentivirus envelope sequence, beginning immediately after the indicated VSV-G residue.

| Name of constructs | Location | Ligand insertion site |
|---|---|---|
| AP1L | N-terminal | RLTRKRGLK-GGGGS-K1 |
| AP1 | N-terminal | RLTRKRGLK-K1 |
| AP2 | Domain I (Lateral domain) | Q10-RLTRKRGLK-K11 |
| AP3 | - | K50-RLTRKRGLK-A51 |
| AP4 | - | K174-RLTRKRGLK-G175 |
| AP5 | Domain III (PH domain) | G193-RLTRKRGLK-E194 |
| AP6 | Domain III (PH domain) | K200-RLTRKRGLK-E201 |
| AP7 | Domain III (PH domain) | E201-RLTRKRGLK-G202 |
| AP8 | Domain I (Lateral domain) | T351-RLTRKRGLK-T352 |
| CS1L | N-terminal | HNMPNDPNRNVDENANANSAYC-GGGGS-K1 |
| CS1 | N-terminal | HNMPNDPNRNVDENANANSAYC-K1 |
| CS2 | Domain I (Lateral domain) | Q10-HNMPNDPNRNVDENANANSAYC-K11 |
| CS3 | - | K50-HNMPNDPNRNVDENANANSAYC-A51 |
| CS4 | - | K174-HNMPNDPNRNVDENANANSAYC-G175 |
| CS5 | Domain III (PH domain) | G193-HNMPNDPNRNVDENANANSAYC-E194 |
| CS6 | Domain III (PH domain) | K200-HNMPNDPNRNVDENANANSAYC-E201 |
| CS7 | Domain III (PH domain) | E201-HNMPNDPNRNVDENANANSAYC-G202 |
| CS8 | Domain I (Lateral domain) | T351-HNMPNDPNRNVDENANANSAYC-T352 |

**Table S2. Nucleotide sequences of IDOL siRNAs used for screening in vitro.** SiNC represents the negative control siRNA sequence. All the candidate siRNAs designed against different regions of human IDOL mRNA (Refseq ID: NM_013262.4).

| Name | Sequence (5'-3') |
|---|---|
| siNC | UUCUCCGAACGUGUCACGU |
| siIDOL-483 | GGAGCCUCAUCUCAUCUUA |
| siIDOL-485 | AGCCUCAUCUCAUCUUACA |
| siIDOL-619 | UUUGGAGACUACAACCAGA |
| siIDOL-622 | GGAGACUACAACCAGAACA |
| siIDOL-904 | GCUUAUCCUGUGGUGCAGA |
| siIDOL-1214 | CCAGGAGGGCUCUGUACAA |
| siIDOL-1406 | GCUGCGAGGAGGAGAUCAA |
| siIDOL-1508 | GUGUGGAGCAUGUCCAGCA |
| siIDOL-1513 | GAGCAUGUCCAGCACGUCU |
| siIDOL-1514 | AGCAUGUCCAGCACGUCUA |

**Table S3. Nucleotide sequences of IDOL shRNAs used for the lentiviral vector** construction. ShNC represents the negative control shRNA sequence and shIDOL is designed based on the sequence of siIDOL-622. The bold letters represent the sequences of negative control siRNA and siIDOL-622.

| Name | | Sequence (5'→ 3') |
|---|---|---|
| shNC | sense | GATCC**GCGACGATCTGCCTAAGAT**TTCAAGAGA**ATCTTAGGCAGATCGTCGC**TTTTTTG |
| | antisense | AATTCAAAAAA**GCGACGATCTGCCTAAGAT**TCTCTTGAA**ATCTTAGGCAGATCGTCGC**G |
| shIDOL | sense | GATCC**GGAGACTACAACCAGAACA**TTCAAGAGA**TGTTCTGGTTGTAGTCTCC**TTTTTTG |
| | antisense | AATTCAAAAAA**GGAGACTACAACCAGAACA**TCTCTTGAA**TGTTCTGGTTGTAGTCTCC**G |

**Table S4. Primer list for construction of lentivrus envelope mutant plasmids**.

| Primer | Sequence (5'→3') |
|---|---|
| HF | CCCAAGCTTACATGTGGTACCGAGCTCGGA |
| PR | CCAATGCATTGGTTCTGCAGCAAAGAGATCCTTATC |
| PF | AAAACTGCAGCCAGATTCCCTGAATGCC |
| NR | TTTTCCTTTTGCGGCCGCACTAGTGCTAGACTGCCA |
| AP1F | TTGACAAGAAAAAGGGGATTGAAGAAGTTCACCATAGTTTTTCC |
| AP1R | CAATCCCCTTTTTCTTGTCAATCTGCAATTCACCCCAATGAATA |
| AP1LF | AAGGGCGGCGGCGGCAGCAAGTTCACCATAGTTTTTCCACAC |
| AP1LR | CTTGCTGCCGCCGCCGCCCTTCAATCCCCTTTTTCTTGTCAA |
| AP2F | TTGACAAGAAAAAGGGGATTGAAGAAAGGAAACTGGAAAAATGT |
| AP2R | CAATCCCCTTTTTCTTGTCAATCTTTGGTTGTGTGGAAAAACTA |
| AP3F | TTGACAAGAAAAAGGGGATTGAAGGCTATTCAAGCAGACGGTTG |
| AP3R | CAATCCCCTTTTTCTTGTCAATCTCTTGTGACTCTTGGGCATTT |
| AP4F | TTGACAAGAAAAAGGGGATTGAAGGGGCTATGTGATTCTAACCT |
| AP4R | CAATCCCCTTTTTCTTGTCAATCTTTTGACCTTATAGTCAGAAT |
| AP5F | TTGACAAGAAAAAGGGGATTGAAGGAGCTATCATCCCTGGGAAA |
| AP5R | CAATCCCCTTTTTCTTGTCAATCTTCCGTCCTCTGAGA |
| AP6F | TTGACAAGAAAAAGGGGATTGAAGGAGGGCACAGGGTTCAGAA |
| AP6R | CAATCCCCTTTTTCTTGTCAATCTCTTTCCCAGGGATGATAGC |
| AP7F | TTGACAAGAAAAAGGGGATTGAAGGGCACAGGGTTCAGAAGTAA |
| AP7R | CAATCCCCTTTTTCTTGTCAATCTCTCCTTTCCCAGGGATGATA |
| AP8F | TTGACAAGAAAAAGGGGATTGAAGACAGAAAGGGAACTGTGGGA |
| AP8R | CAATCCCCTTTTTCTTGTCAATCTGGTAGTTCCACTGATCATTC |
| CS1F | AACCGAAATGTAGATGAAAATGCTAATGCCAACAGTGCTAAGTTCAC CATAGTTTTTCC |
| CS1R | ATTTTCATCTACATTTCGGTTTGGGTCATTTGGCATATTGTGGCAATTC ACCCCAATGA |
| CS1LF | GCTGGCGGCGGCGGCAGCAAGTTCACCATAGTTTTTCCACAC |
| CS1LR | CTTGCTGCCGCCGCCGCCAGCACTGTTGGCATTAGCATTTTC |
| CS2F | AACCGAAATGTAGATGAAAATGCTAATGCCAACAGTGCTAAAGGAAA |

| | |
|---|---|
| | CTGGAAAAATGT |
| CS2R | ATTTTCATCTACATTTCGGTTTGGGTCATTTGGCATATTGTGTTGGTTG TGTGGAAAAA |
| CS3F | AACCGAAATGTAGATGAAAATGCTAATGCCAACAGTGCTGCTATTCA AGCAGACGGTTG |
| CS3R | ATTTTCATCTACATTTCGGTTTGGGTCATTTGGCATATTGTGCTTGTGA CTCTTGGGC |
| CS4F | CCAAACCGAAATGTAGATGAAAATGCTAATGCCAACAGTGCTGGGCT ATGTGATTCTAA |
| CS4R | ATTTTCATCTACATTTCGGTTTGGGTCATTTGGCATATTGTGTTTGACC TTATAGTCAG |
| CS5F | CCAAACCGAAATGTAGATGAAAATGCTAATGCCAACAGTGCTGAGCT ATCATCCCTGG |
| CS5R | ATTTTCATCTACATTTCGGTTTGGGTCATTTGGCATATTGTGTCCGTCC TCTGAGAAGA |
| CS6F | AACCGAAATGTAGATGAAAATGCTAATGCCAACAGTGCTGAGGGCAC AGGGTTCAGAAG |
| CS6R | ATTTTCATCTACATTTCGGTTTGGGTCATTTGGCATATTGTGCTTTCCC AGGGATGATA |
| CS7F | CCAAACCGAAATGTAGATGAAAATGCTAATGCCAACAGTGCTGGCAC AGGGTTCAGAAG |
| CS7R | ATTTTCATCTACATTTCGGTTTGGGTCATTTGGCATATTGTGCTCCTTTC CCAGGGAT |
| CS8F | CCAAACCGAAATGTAGATGAAAATGCTAATGCCAACAGTGCTACAGA AAGGGAACTGTG |
| CS8R | ATTTTCATCTACATTTCGGTTTGGGTCATTTGGCATATTGTGGGTAGTT CCACTGATCA |

**Table S5. Primers for qRT-PCR.**

| Gene | Primer sequence (5’-3’) | |
|---|---|---|
| | Forward | Reverse |
| Human IDOL | CACCCAGTCAGGAAAG | GTTATCGCTCGGTAGAG |
| Human GAPDH | TGACAACAGCCTCAAGAT | ACCACCCTGTTGCTGTAGCC |
| Mouse IDOL | CCCAGTCAGGAAAGAAT | GGTGATGGCTCGGTAG |
| Mouse β-actin | GTGACGTTGACATCCGTAAAGA | GCCGGACTCATCGTACTCC |
